# Reconstruction of time-consistent species trees

**DOI:** 10.1186/s13015-020-00175-0

**Published:** 2020-08-20

**Authors:** Manuel Lafond, Marc Hellmuth

**Affiliations:** 1 Department of Computer Science, Université de Sherbrooke, 2500 Boul. de l’Université, Sherbrooke, J1K 2R1 Canada; 2 School of Computing, University of Leeds, E C Stoner Building, Leeds, LS2 9JT UK

**Keywords:** Tree reconciliation, Gene evolution, Species evolution, Horizontal gene transfer, Time-consistency, Polynomial-time algorithm

## Abstract

**Background:**

The history of gene families—which are equivalent to event-labeled gene trees—can to some extent be reconstructed from empirically estimated evolutionary event-relations containing pairs of orthologous, paralogous or xenologous genes. The question then arises as whether inferred event-labeled gene trees are “biologically feasible” which is the case if one can find a species tree with which the gene tree can be reconciled in a time-consistent way.

**Results:**

In this contribution, we consider event-labeled gene trees that contain speciations, duplications as well as horizontal gene transfer (HGT) and we assume that the species tree is unknown. Although many problems become NP-hard as soon as HGT and time-consistency are involved, we show, in contrast, that the problem of finding a time-consistent species tree for a given event-labeled gene can be solved in polynomial-time. We provide a cubic-time algorithm to decide whether a “time-consistent” species tree for a given event-labeled gene tree exists and, in the affirmative case, to construct the species tree within the same time-complexity.

## Background

Genes collectively form the organism’s genomes and can be viewed as “atomic” units whose evolutionary history forms a tree. The history of species, which is also a tree, and the history of their genes is intimately linked, since the gene trees evolve along the species tree. A detailed evolutionary scenario, therefore, consists of a gene tree, a species tree and a reconciliation map $\mu$ that describes how the gene tree is embedded into the species tree.

A reconciliation map assigns vertices of the gene tree to the vertices or edges in the species in such a way that (partial) ancestor relations given by the genes are preserved by the map $\mu$. This gives rise to three important events that may act on the genes through evolution: *speciation*, *duplication*, and *horizontal gene transfer (HGT)* [1, 2]. Inner vertices of the species tree represent speciation events. Hence, vertices of the gene tree that are mapped to inner vertices in the species tree underlay a speciation event and are transmitted from the parent species into the daughter species. If two copies from a single ancestral gene are formed and reside in the same species, then a duplication event happened. Contrary, if one of the copies of a gene “jumps” into a different branch of the species tree, then a HGT event happened. The latter can be annotated in the gene tree by associating a label to the edge that points from the horizontal transfer event to the transferred copy [3–7]. Since both HGT and duplication events occur in between different speciation events, such vertices of the gene trees are usually mapped to the edges of the species tree. The events speciation, duplication, and HGT classify pairs of genes as orthologs, paralogs and xenologs, respectively [2].

To some extent, these relations can be estimated directly from sequence data using a variety of algorithmic approaches that are based on the pairwise best match criterion [8–11] and hence do not require any *a priori* knowledge of the topology of either the gene tree or the species tree. Practical workflows for orthology assignment directly use pairwise best matches as an initial estimate of orthologous gene pairs. Many of the commonly used methods for orthology-identification, such as OrthoMCL [12], ProteinOrtho [13, 14], OMA [15], or eggNOG [16], belong to this class, see also [17, 18]. While best match heuristics have been very successful as approximations of the orthology relation [19, 20], no comparable approach to extract the xenology relations directly from (dis)similarity data has been devised to-date. Nevertheless, there are several methods to detect xenologs in a genome that use sequence features rather than phylogenetic reconstructions, see e.g. [21–26].

Provided that such event-relations are available, one can infer the history of event-labeled gene trees without HGT [27–32] or with HGT [5, 7, 33]. Moreover, depending on the quality and biological feasibility of the reconstructed event-labeled gene trees, a species trees can also be reconstructed [3, 34, 35]. This line of research, in particular, has been very successful for the reconstruction of event-labeled gene trees and species trees based solely on the information of orthologous and paralogous gene pairs [36]. Note that in practice, inferred event-relations are likely to contain errors. However, characterizing relations that correspond to a valid history is a crucial step in devising error-correction algorithms, as this may lead to practical heuristic approaches to modify noisy event-relations into valid ones.

In this paper, we assume that the gene tree *T* and the types of evolutionary events on *T* are known. For an event-labeled gene tree to be biologically feasible there must be a putative “true” history that can explain the inferred gene tree. However, in practice it is not possible to observe the entire evolutionary history as e.g. gene losses eradicate the entire information on parts of the history. Therefore, the problem of determining whether an event-labeled gene tree is biologically feasible is reduced to the problem of finding a valid reconciliation map, also known as DTL-scenario [37–39]. The aim is then to find the unknown species tree *S* and reconciliation map between *T* and *S*, if one exists. Not all event-labeled gene trees *T*, however, are biologically feasible in the sense that that there exists a species tree *S* such that *T* can be reconciled with *S*. In the absence of HGT, biologically feasibility can be characterized in terms of “informative” triplets (rooted binary trees on three leaves) that are displayed by the gene trees [35]. In the presence of HGT, such triplets give at least necessary conditions for a gene tree being biologically feasible [3].

A particular difficulty that occurs in the presence of HGT is that gene trees with HGT must be mapped to species trees only in such a way that genes do not travel back in time. To be more precise, the ancestor ordering of the vertices in a species tree give rise to a relative timing information of the species within the species trees. Within this context, speciation and duplication events can be considered as a vertical evolution, that is, the genetic material is transferred “forward in time”. In contrast, HGT literally yield horizontal evolution, that is, genetic material is transferred such that a gene and its transferred copy coexist. Nøjgaard et al. [4] introduced an axiomatic framework for time-consistent reconciliation maps and characterize for given event-labeled gene trees *T* and a *given* species tree *S* whether there exists a time-consistent reconciliation map or not. This characterization resulted in an $O(|V|\log |W|)$-time algorithm to construct a time-consistent reconciliation map if one exists, where *V* and *W* are the vertex sets of *T* and *S*, respectively.

However, one of the crucial open questions that were left open within this context is as follows: *For a given event-labeled gene tree that contains speciation and duplication vertices and HGT edges, does there exist a polynomial-time algorithm to reconstruct the *unknown* species tree together with a time-consistent reconciliation map, if one exists?*

In this contribution, we show that the answer to this problem is affirmative and provide an $O(n^3)$ time algorithm, with *n* being the number of leaves of *T*, that allows us to verify whether there is a time-consistent species *S* for the event-labeled gene tree and, in the affirmative case, to construct *S*.

We note in passing that there could be exponentially many species trees, for each of them there may be a time-consistent reconciliation map or not for a given event-labeled gene tree. Moreover, many types of reconciliation problems become NP-hard as soon as HGT and time-consistency are involved, see e.g. [37, 40–46]. In contrast, we show that the problem of finding a time-consistent species tree for a given event-labeled gene tree can be solved in polynomial-time.

This paper is organized as follows. We first provide a short survey of preliminary results that have been establised so far and therein, provide the concepts and basic notation we need including important results on gene and species tree, reconciliation maps and time-consistency. We then proceed in Section "Gene tree consistency" (GTC) to formally introduce the problem of finding a time-consistent species for a given event-labeled gene tree. As a main result, we will see that it suffices to start with a fully unresolved species tree that can then be stepwisely extended to a binary species tree to obtain a solution to the GTC problem, provided a solution exists. In Section "An algorithm for the GTC problem", we provide a solution to the GTC problem. For the design of this algorithm, we will utilize an auxiliary directed graph $A({T},{S})$ based on a given event-labeled gene tree *T* and a given species tree *S*. This type of graph was established in [4]. The authors showed that there is time-consistent map between *T* and *S* if and only if $A({T},{S})$ is acyclic. Our algorithm either reconstructs a species tree *S* based on the informative triplets that are displayed by the gene trees and that makes this graph $A({T},{S})$ eventually acyclic or that returns that no solution exists. The strategy of our algorithm is to construct $A({T},{S})$ starting with *S* being a fully unresolved species tree and stepwisely resolve this tree in a way that it “agrees” with the informative triplets and reduces the cycles in $A({T},{S})$.

Since the material is rather extensive (many of the proofs use elementary graph theory but are very technical) we subdivided the presentation in a main narrative text explaining the main results and a second technical part (see Appendix: “Proof of results”) collecting the proofs of the main results as well as additional technical results.

## Short survey of existing results

### Notation and basic definitions

Unless stated otherwise, all graphs in this work are assumed to be directed without explicit mention. For a graph *G*, the subgraph induced by $X \subseteq V(G)$ is denoted *G*[*X*]. For a subset $Q \subseteq V(G)$, we write $G - Q = G[V(G) \setminus Q]$. We will write (*a*, *b*) and *ab* for the edges that link $a,b\in V(G)$ of directed, resp., undirected graphs.

All trees in this work are rooted and edges are directed away from the root. Given a tree *T*, a vertex $v \in V(T)$ is a *leaf* if *v* has out-degree 0, and an *internal vertex* otherwise. We write $L(T)$ to denote the set of leaves of *T*. A *star tree* is a tree with only one internal vertex that is adjacent to the leaves.

We write $x \preceq _{T} y$ if *y* lies on the unique path from the root to *x*, in which case *y* is called an ancestor of *x* and *x* is called a descendant of *y*. We may also write $y \succeq _{T} x$ instead of $x \preceq _{T} y$. We use $x \prec _T y$ for $x \preceq _{T} y$ and $x \ne y$. In the latter case, *y* is a *strict ancestor* of *x*. If $x \preceq _{T} y$ or $y \preceq _{T} x$ the vertices *x* and *y* are *comparable* and, otherwise, *incomparable*. If (*x*, *y*) is an edge in *T*, and thus, $y \prec _{T} x$, then *x* is the *parent* of *y* and *y* the *child* of *x*. We denote with $\mathrm {ch}(x)$ the set of all children of *x*. It will be convenient for the discussion below to extend the ancestor relation $\preceq _T$ on *V* to the union of the edge and vertex sets of *T*. More precisely, for a vertex $x\in V(T)$ and an edge $e=(u,v)\in E(T)$ we put $x \prec _T e$ if and only if $x\preceq _T v$ and $e \prec _T x$ if and only if $u\preceq _T x$. For edges $e=(u,v)$ and $f=(a,b)$ in *T* we put $e\preceq _T f$ if and only if $v \preceq _T b$.

For a subset $X \subseteq V(T)$, the *lowest common ancestor* ${\text {lca}}_{T}(X)$ is the unique $\preceq _T$-minimal vertex that is an ancestor of all vertices in *X* in *T*. For simplicity, we often write ${\text {lca}}_T(x,y)$ instead of ${\text {lca}}_T(\{x,y\})$.

A vertex is *binary* if it has 2 children, and *T* is *binary* if all its internal vertices are binary. A *cherry* is an internal vertex whose children are all leaves (note that a cherry may have more than two children). A tree *T* is *almost binary* if its only non-binary vertices are cherries. For $v \in V(T)$, we write *T*(*v*) to denote the subtree of *T* rooted at *v* (i.e. the tree induced by *v* and its descendants).

A *rooted triplet*, or *triplet* for short, is a binary tree with three leaves. We write *ab*|*c* to denote the unique triplet on leaf set $\{a,b,c\}$ in which the root is ${\text {lca}}(a,c) = {\text {lca}}(b,c)$. We say that a tree *T* *displays* a triplet *ab*|*c* if $a,b,c \in L(T)$ and ${\text {lca}}_T(a, b) \prec {\text {lca}}_T(a,c) = {\text {lca}}_T(b,c)$. We write *rt*(*T*) to denote the set of rooted triplets that *T* displays. Given a set of triplets *R*, we say that *T* *displays* *R* if $R \subseteq rt(T)$. A set of triplets *R* is *compatible*, if there is a tree that displays *R*. We also say that *T* *agrees* with *R* if, for every $ab|c \in R$, $ac|b \notin rt(T)$ and $bc|a \notin rt(T)$.

#### Remark 1

The term “agree” is more general than the term “display” and “compatible”, i.e., if *T* displays *R* (and thus, *R* is compatible), then *T* must agree with *R*. The converse, however, is not always true. To see this, consider the star tree *T* , i.e., $rt(T) = \emptyset$, and let $R=\{ab|c,bc|a\}$. It is easy to verify that *R* is incompatible since there cannot be any tree that displays both triplets in *R*. However, the set *R* agrees with *T*.

We will consider rooted trees $T=(V,E)$ from which particular edges are removed. Let $\mathcal {E}_T \subseteq E$ and consider the forest $T_{\mathcal {\overline{E}}}{:}{=}(V,E\setminus \mathcal {E}_T)$. We can preserve the order $\preceq _T$ for all vertices within one connected component of $T_{\mathcal {\overline{E}}}$ and define $\preceq _{T_{\mathcal {\overline{E}}}}$ as follows: $x\preceq _{T_{\mathcal {\overline{E}}}}y$ iff $x\preceq _{T}y$ and *x*, *y* are in same connected component of $T_{\mathcal {\overline{E}}}$. Since each connected component $T'$ of $T_{\mathcal {\overline{E}}}$ is a tree, the ordering $\preceq _{T_{\mathcal {\overline{E}}}}$ also implies a root $\rho _{T'}$ for each $T'$, that is, $x\preceq _{T_{\mathcal {\overline{E}}}} \rho _{T'}$ for all $x\in V(T')$. If $L(T_{\mathcal {\overline{E}}})$ is the leaf set of $T_{\mathcal {\overline{E}}}$, we define $L_{T_{\mathcal {\overline{E}}}}(x) = \{y\in L(T_{\mathcal {\overline{E}}}) \mid y\preceq _{T_{\mathcal {\overline{E}}}} x\}$ as the set of leaves in $T_{\mathcal {\overline{E}}}$ that are reachable from *x*. Hence, all $y\in L_{T_{\mathcal {\overline{E}}}}(x)$ must be contained in the same connected component of $T_{\mathcal {\overline{E}}}$. We say that the forest $T_{\mathcal {\overline{E}}}$ displays a triplet *r*, if *r* is displayed by one of its connected components. Moreover, $rt(T_{\mathcal {\overline{E}}})$ denotes the set of all triplets that are displayed by the forest $T_{\mathcal {\overline{E}}}$.

The *restriction* $T|_X$ of *T* to $X\subseteq L(T)$, is the tree with leaf set *X* that is obtained from *T* by first taking the minimal subtree of *T* with leaf set *X* and then suppressing all vertices of degree two with the exception of the root of $T|_X$.

### Gene and species trees

Let $\Gamma$ and $\Sigma$ be a set of genes and a set of species, respectively. Moreover, we assume we know the gene-species association, i.e., a surjective map $\sigma : \Gamma \rightarrow \Sigma$. A *species tree* is a tree *S* such that $L(S) \subseteq \Sigma$. A *gene tree* is a tree *T* such that $L(T) \subseteq \Gamma$. Note that $\sigma (l)$ is defined for every leaf $l \in L(T)$. We extend $\sigma$ to vertices of *T* and put $\sigma _T(v) = \{\sigma (l) :l \in L(T(v))\}$. We may drop the *T* subscript whenever there is no risk of confusion. For $\mathcal {E}_T \subseteq E$, the $\sigma$ notation also extends to $\sigma _{T_{\mathcal {\overline{E}}}}$, i.e. we write $\sigma _{T_{\mathcal {\overline{E}}}}(u){:}{=}\{ \sigma (l) :{:} l \in L_{T_{\mathcal {\overline{E}}}}(u) \}$. We emphasize that species and gene trees need not to be binary, which are used here to model incomplete knowledge of the exact gene phylogenies.

Given a gene tree *T*, we assume knowledge of a labeling function ${t : V(T) \cup E(T) \rightarrow \{\odot , \mathfrak {s}, \mathfrak {d}, \mathfrak {t}\} \cup \{0, 1\}}$. We require that $t(v) \in \{\odot , \mathfrak {s}, \mathfrak {d}, \mathfrak {t}\}$ for all ${v \in V(T)}$ and $t(e) \in \{0,1\}$ for all $e \in E(T)$. Each symbol represents a different vertex type: $\odot$ are leaves, $\mathfrak {s}$ are speciations, $\mathfrak {d}$ are duplications and $\mathfrak {t}$ indicates vertices from which a horizontal gene transfer started. Edges labeled by 1 represent horizontal transfers and edges labeled by 0 represent vertical descent. Here, we always assume that only edges (*x*, *y*) for which $t(x) = \mathfrak {t}$ might be labeled as transfer edge; $t(x,y)=1$. We let $\mathcal {E}_T = \{e \in E(T) :t(e) = 1\}$ be the set of transfer edges. We also require that $t(u) = \odot$ if and only if $u \in L(T)$.

We write $(T; t, \sigma )$ to denote a gene tree *T* labeled by *t* having gene-species mapping $\sigma$.

In what follows we will only consider labeled gene trees $(T;t,\sigma )$ that satisfy the following three axioms:

(O1): Every internal vertex *v* has out-degree at least 2.
(O2): Every transfer vertex *x* has at least one transfer edge $e=(x,v)$ labeled $t(e)=1$, and at least one non-transfer edge $f=(x,w)$ labeled $t(f)=0$;
(O3): (a) If $x\in V(T)$ is a speciation vertex with children $v_1,\dots ,v_k$, $k\ge 2$, then $\sigma _{T_{\mathcal {\overline{E}}}}(v_i) \cap \sigma _{T_{\mathcal {\overline{E}}}}(v_j) =\emptyset$, $1\le i<j\le k$;
  (b) If $$(x,y) \in \mathcal {E}_T$$, then $$\sigma _{T_{\mathcal {\overline{E}}}}(x)\cap \sigma _{T_{\mathcal {\overline{E}}}}(y) = \emptyset .$$

These conditions are also called “observability-axioms” and are exhaustively discussed in [3] and [4]. We repeat here shortly the arguments to justify Condition (O1–O3). Usually the considered labeled gene trees are obtained from genomic sequence data. Condition (O1) ensures that every inner vertex leaves a historical trace in the sense that there are at least two children that have survived. If this were not the case, we would have no evidence that vertex *v* ever exist. Condition (O2) ensures that for an HGT event a historical trace remains of both the transferred and the non-transferred copy. Furthermore, there is no clear evidence for a speciation vertex *v* if it does not “separate” lineages, which is ensured by Condition (O3.a). Finally (O3.b) is a simple consequence of the fact that if a transfer edge (*x*, *y*) in the gene tree occurred, then the species *X* and *Y* that contain *x* and *y*, respectively, cannot be ancestors of each other, as otherwise, the species *X* and *Y* would not coexist (cf. [4, Prop. 1]).

We emphasize that Lemma 1 in [4] states that the leaf set $L_1,\dots ,L_k$ of the connected components $T_1,\dots ,T_k$ of $T_{\mathcal {\overline{E}}}$ forms a partition of *L*(*T*), which directly implies that $\sigma _{T_{\mathcal {\overline{E}}}}(x) \ne \emptyset$ for all $x\in V(T)$.

#### Remark 2

In what follows we always assume that a species tree satisfies (O1). This condition is used to ensure that every species tree is a so-called *phylogenetic* tree [47].

However, as it is possible that gene duplications and losses predate the first speciation event, we may model the species tree *S* as a *planted* tree, that is, there is an additional vertex $x\succ \rho _S = {\text {lca}}(L(S))$ with unique child $\rho _S$. However, for our technical results below, this planted root is not of further importance.

By slight abuse of notation and to keep the upcoming proofs simple, we still call $\rho _S = {\text {lca}}(L(S))$ the *root* of *S* and the parent of $\rho _S$ in *S*, the *planted root* of *S*.

### Reconciliation maps and speciation triplets

The “embedding” of the gene tree into the species tree is formalized by a *reconciliation map from* $(T;t,\sigma )$*to* *S*, that is, a map $\mu : V(T) \rightarrow V(S)\cup E(S)$ that satisfies the following constraints for all $x\in V(T)$:

(M1): *Leaf constraint.* If $x\in \Gamma$, then $\mu (x)=\sigma (x)$.
(M2): *Event constraint.*

(i) If $$t(x)=\mathfrak {s}$$, then $$\mu (x) = {\text {lca}}_S(\sigma _{T_{\mathcal {\overline{E}}}}(x))$$.
(ii) If $t(x) \in \{\mathfrak {d}, \mathfrak {t}\}$, then $\mu (x)\in E(S)$.
(iii) If $t(x)=\mathfrak {t}$ and $(x,y)\in \mathcal {E}_T$, then $\mu (x)$ and $\mu (y)$ are incomparable in *S*.
(iv) If $t(x)=\mathfrak {s}$, then $\mu (u)$ and $\mu (v)$ are incomparable in *S* for all distinct $u,v\in \mathrm {ch}(x)$.

(M3): *Ancestor constraint.* Let $x,y\in V(T)$ with $x\prec _{T_{\mathcal {\overline{E}}}} y$. Note, the latter implies that the path connecting *x* and *y* in *T* does not contain transfer edges. We distinguish two cases:

(i) If $$t(x),t(y)\in \{\mathfrak {d}, \mathfrak {t}\}$$, then $$\mu (x)\preceq _S \mu (y)$$,
(ii) otherwise, i.e., at least one of *t*(*x*) and *t*(*y*) is a speciation $\mathfrak {s}$, $\mu (x)\prec _S\mu (y)$.

We call $\mu$ the *reconciliation map* from $(T;t,\sigma )$ to *S*. The provided definition of a reconciliation map coincides with the one as given in [3, 4, 48] and is a natural generalization of the maps as in [35, 37, 38, 49] for the case where no HGT took place.

The question arises when for a given gene tree $(T;t,\sigma )$ a species tree *S* together with a reconciliation map $\mu$ from $(T;t,\sigma )$ to *S* exists. An answer to this question is provided by

#### Definition 1

(*Informative triplets*) Let $(T;t,\sigma )$ be an event-labeled gene tree. The set $\mathcal {R}(T;t,\sigma )$ is the set of triplets $\sigma (a)\sigma (b)|\sigma (c)$ where $\sigma (a),\sigma (b),\sigma (c)$ are pairwise distinct and either

1. *ab*|*c* is a triplet displayed by $T_{\mathcal {\overline{E}}}$ and $t({\text {lca}}_{T_{\mathcal {\overline{E}}}} (a, b, c)) = \mathfrak {s}$ or
2. $a, b \in L(T_{\mathcal {\overline{E}}}(x))$ and $c \in L(T_{\mathcal {\overline{E}}}(y))$ for some transfer edge (*x*, *y*) or (*y*, *x*) in $\mathcal {E}_T$

#### Theorem 1

([3]) *Let* $(T;t,\sigma )$*be a labeled gene tree. Then, there is a species tree* *S* *together with a reconciliation map* $\mu$*from* $(T;t,\sigma )$*to* *S* *if and only if* $\mathcal {R}(T;t,\sigma )$*is compatible. In this case, every species tree* *S* *that displays* $\mathcal {R}(T;t,\sigma )$*can be reconciled with* $(T;t,\sigma )$.

*Moreover, there is a polynomial-time algorithm that returns a species tree* *S* *for* $(T;t, \sigma )$*together with a reconciliation map* $\mu$*in polynomial time, if one exists and otherwise, returns that there is no species tree for* $(T;t, \sigma )$.

It has been shown in [3], that if there is any reconciliation map from $(T;t,\sigma )$ to *S*, then there is always a reconciliation map $\mu$ that additionally satisfies for all $u\in V(T)$ with $t(u)\in \{\mathfrak {d},\mathfrak {t}\}$:

$$\begin{aligned} \mu (u) = (v,{\text {lca}}_S(\sigma _{T_{\mathcal {\overline{E}}}}(u)))\in E(S) \end{aligned}$$

where *v* denotes the unique parent of ${\text {lca}}_S(\sigma _{T_{\mathcal {\overline{E}}}}(u))$ in *S*. As a consequence, we consider the following simplification.

#### Definition 2

The *LCA-map* $\widehat{\mu }_{T,S}:V(T) \rightarrow V(S)$ associates every vertex $v \in V(T)$ to the lowest common ancestor of $\sigma _{T_{\mathcal {\overline{E}}}}(v)$, i.e., $\widehat{\mu }_{T, S}(v) {:}\,{=}\,lca_{S}(\sigma _{T_{\mathcal {\overline{E}}}}(v))$

#### Remark 3

Note that if *v* is a leaf of *T*, we have $\widehat{\mu }_{T, S}(v) = \sigma (v)$. Moreover, the LCA-map $\widehat{\mu }_{T, S}$ always exists and is uniquely defined, although there might be no reconciliation map from $(T;t,\sigma )$ to *S*.

We may write $\widehat{\mu }, \widehat{\mu }_{T}$ or $\widehat{\mu }_{S}$ if *T* and/or *S* are clear from the context.

Compatibility of $\mathcal {R}(T; t,\sigma )$ provides a necessary condition for the existence of *biologically feasible* reconciliation, i.e., maps that are additionally time-consistent. To be more precise:

#### Definition 3

(*Time map*) The map $\tau _T:V(T) \rightarrow {\mathbb {R}}$ is called a *time map* for the rooted tree *T* if $x\prec _T y$ implies $\tau _T(x)>\tau _T(y)$ for all $x,y\in V(T)$.

#### Definition 4

(*Time-consistent*) A reconciliation map $\mu$ from $(T;t,\sigma )$ to *S* is *time-consistent* if there are time maps $\tau _T$ for *T* and $\tau _S$ for *S* satisfying the following conditions for all $u\in V(T)$:

(B1): If $$t(u) \in \{\mathfrak {s}, \odot \}$$, then $$\tau _T(u) = \tau _S(\mu (u))$$.
(B2): If $t(u)\in \{\mathfrak {d},\mathfrak {t}\}$ and, thus $\mu (u)=(x,y)\in E(S)$, then $\tau _S(y)>\tau _T(u)>\tau _S(x)$.

If a time-consistent reconciliation map from $(T;t,\sigma )$ to *S* exists, we also say that *S* is a *time-consistent species tree for* $(T;t,\sigma )$.

**Fig. 1:**
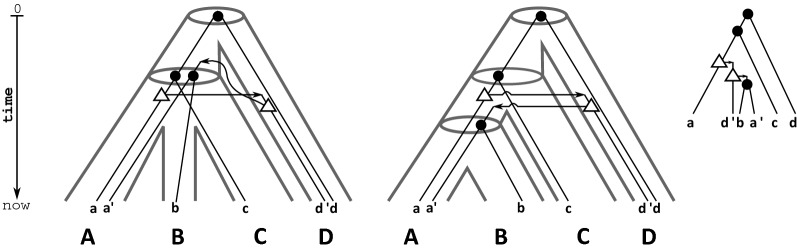
Taken from [3, Fig. 4]. From the binary gene tree $(T;t,\sigma )$ (right) we obtain the species triplets $\mathcal {R}(T;t,\sigma ) = \{AB|D,AC|D\}$ . Note, vertices *v* of *T* with $t(v)=\mathfrak {s}$ and $t(v)=\mathfrak {t}$ are highlighted by “$\bullet$” and “$\triangle$”, respectively. Transfer edges are marked with an “arrow”. Shown are two (tube-like) species trees (left and middle) where planted roots are omitted that display $\mathcal {R}(T;t,\sigma )$. Thus, Theorem 1 implies that for both trees a reconciliation map from $(T;t,\sigma )$ exists. The respective reconciliation maps for $(T;t,\sigma )$ and the species tree are given implicitly by drawing $(T;t,\sigma )$ within the species tree. The left species tree *S* is least resolved for $\mathcal {R}(T;t,\sigma )$. The reconciliation map from $(T;t,\sigma )$ to *S* is unique, however, not time-consistent. Thus, no time-consistent reconciliation between *T* and *S* exists at all. On the other hand, for *T* and the middle species tree (that is a refinement of *S*) there is a time-consistent reconciliation map

Figure 1 gives an example for two different species trees that both display $\mathcal {R}(T;t,\sigma )$ for which only one admits a time-consistent reconciliation map. Further examples can be found in [3, 4].

### Auxiliary graph construction

When the species tree is known, one can efficiently determine whether a time-consistent map for a given gene *G* and species tree *S* exists. We will use an auxiliary graph as defined in [4], and will investigate the structure of this graph in the remaining part of this section. Intuitively, this graph exhibits timing information between genes and species. That is, the gene tree events allow us to determine constraints of the form “*x* must have existed before *y*”, and each such constraint is represented by an arc from *x* to *y*. As it turns out, the absence of cycles in the resulting graph is necessary and sufficient to determine the existence of a time-consistent map for a given event-labeled gene tree and a given species tree.

Let $(T;t,\sigma )$ be a labeled gene tree and *S* be a species tree. Let $A({T},{S})$ be the graph with vertex set $V(A({T},{S})) = V(T) \cup V(S)$, and edge set $E(A({T},{S}))$ constructed from four sets as follows:

(A1): For each $(u, v) \in E(T)$, we have $(u', v') \in E(A({T},{S}))$, where
  $$\begin{aligned} u' = {\left\{ \begin{array}{ll} \widehat{\mu }(u) &{}\hbox { if}\ t(u) \in \{\odot , \mathfrak {s}\} \\ u &{}\text{ otherwise } \end{array}\right. } \end{aligned}$$
  and
  $$\begin{aligned} v' = {\left\{ \begin{array}{ll} \widehat{\mu }(v) &{}\hbox { if}\ t(v) \in \{\odot , \mathfrak {s}\} \\ v &{}\text{ otherwise } \end{array}\right. } \end{aligned}$$
(A2): For each $(x, y) \in E(S)$, we have $(x, y) \in E(A({T},{S}))$
(A3): For each *u* with $t(u) \in \{\mathfrak {d}, \mathfrak {t}\}$, we have $(u, \widehat{\mu }(u)) \in E(A({T},{S}))$
(A4): for each $(u, v) \in \mathcal {E}_{T}$, we have $(lca_S(\widehat{\mu }(u), \widehat{\mu }(v)), u) \in E(A({T},{S}))$

We are aware of the fact that the graph $A({T},{S})$ heavily depends on the event-labeling *t* and the species assignment $\sigma$ of the gene tree $(T;t,\sigma )$. However, to keep the notation simple we will write, by slight abuse of notation, $A({T},{S})$ instead of the more correct notation $A({(T;t,\sigma )},{S})$. The $A({T},{S})$ graph has four types of edges, and we shall refer to them as the A1, A2, A3-and A4-edges, respectively. We note for later reference that if (*x*, *y*) is an A1-edge such that $x, y \in V(S)$, then we must have $y \preceq _S x$ which follows from the definition of $\widehat{\mu }_{T,S}$ and the fact that $\sigma _{T_{\mathcal {\overline{E}}}}(y) \subseteq \sigma _{T_{\mathcal {\overline{E}}}}(x)$.

We emphasize, that the definition of $A({T},{S})$ slightly differs from the one provided in [4]. While Properties (A2), (A3) and (A4) are identical, (A1) was defined in terms of a reconciliation map $\mu$ from $(T;t,\sigma )$ to *S* in [4]. To be more precise, in [4] it is stated $u' = \mu (u)$ and $v' = \mu (v)$ for speciation vertices or leaves *u* and *v* instead of $u' = \widehat{\mu }(u)$ and $v' = \widehat{\mu }(v)$, respectively. However, Condition (M1) and (M2.i) imply that $\mu (u)=\widehat{\mu }(u)$ and $\mu (v)=\widehat{\mu }(v)$ provided $\mu$ exists. In other words, the definition of $A({T},{S})$ here and in [4] are identical, in case a reconciliation map $\mu$ exists.

Since we do not want to restrict ourselves to the existence of a reconciliation map (a necessary condition is provided by Theorem 1) we generalized the definition of $A({T},{S})$ in terms of $\widehat{\mu }$ instead.

For later reference, we summarize the latter observations in the following remark.

#### Remark 4

The graph $A({T},{S})$ does not explicitly depend on a reconciliation map. That is, even if there is no reconciliation map at all, $A({T},{S})$ is always well-defined.

The graph $A({T},{S})$ will be utilized to characterize gene-species tree pairs that admit a time-consistent reconciliation map. For a given gene tree $(T;t,\sigma )$ and a given species tree *S*, the existence of a time-consistent reconciliation map can easily be verified in polynomial time.

#### Theorem 2

([3, 4]) *Let* $(T;t,\sigma )$*be a labeled gene tree and* *S* *be a species tree. Then* *T* *admits a time-consistent reconciliation map with* *S* *if and only if* *S* *displays every triplet of* $\mathcal {R}(T; t,\sigma )$*and* $A({T},{S})$*is acyclic.*

*Recognition and reconstruction of a time-consistent reconciliation map can then be done in* $O(|V(T)|\log (|V(S)|))$*time.*

In Appendix: "Lemma 3 and Proof of Lemma 1", we provide Lemma 3 that is rather technical, but essentially implies the following

#### Remark 5

If *S* has a leaf that forms a self-loop in $A({T},{S})$, then we may immediately discard *S* as it cannot have a solution (since any refinement will have this self-loop). For the rest of the section, we will therefore assume that no leaf of *S* belongs to a self-loop.

## Gene tree consistency

The main question of interest of this work is to determine whether a species tree *S* exists at all for a labeled gene tree *T*. Here, we solve a slightly more general problem: the one of refining a given almost binary species tree *S* so that *T* can be reconciled with it. The idea of our approach is to refine *S* in a step-wise manner using *extension* moves, as defined below.

### Definition 5

(*Extension*) Let *x* be a vertex of a tree *T* with $\mathrm {ch}(x) = \{x_1, \ldots , x_k\}$, $k\ge 3$ and suppose that $X' \subset \mathrm {ch}(x)$ is a strict subset of $\mathrm {ch}(x)$.

Then, the $(x, X')$*extension* modifies *T* to the tree $T_{x,X'}$ as follows: If $|X'| \le 1$, then put $T_{x,X'}=T$. Otherwise, remove the edges $(x, x')$ for each $x' \in X'$ from *T* and add a new vertex *y* together with the edges (*x*, *y*) and $(y,x')$ for all $x'\in X'$ to obtain the tree $T_{x,X'}$.

Given two trees *T* and $T'$, we say that $T'$ is a *refinement* of *T* if there exists a sequence of extensions that transforms *T* into $T'$.

The gene tree consistency (GTC) problem:

Given: A labeled gene tree $(T;t,\sigma )$ and an almost binary species tree *S*.

Question: Does there exist a refinement $S^*$ of *S* that displays $\mathcal {R}(T; t,\sigma )$ and such that $A({T},{S^*})$ is acyclic?

It is easy to see that the problem of determining the existence of a species tree *S* that displays $\mathcal {R}(T; t,\sigma )$ and such that $A({T},{S})$ is acyclic is a special case of this problem. Indeed, it suffices to provide a star tree *S* as input to the GTC problem, since every species tree is then a refinement of *S*.

### Definition 6

A species tree $S^*$*is a solution* to a given GTC instance $((T;t,\sigma ), S)$ if $S^*$ is a refinement of *S*, $S^*$ displays $\mathcal {R}(T; t,\sigma )$ and $A({T},{S^*})$ is acyclic.

We first show that, as a particular case of the following lemma, one can restrict the search to binary species trees (even if *T* is non-binary).

### Lemma 1

*Let* $((T;t,\sigma ), S)$*be a GTC instance and assume that a species tree* $\widehat{S}$*is a solution to this instance. Then any refinement* $S^*$*of* $\widehat{S}$*is also a solution to* $((T;t,\sigma ), S)$.

### Proof

See Appendix: "Lemma 3 and Proof of Lemma 1". $\square$

This shows that we can restrict our search to binary species trees, and we may only require that it *agrees* with $\mathcal {R}(T;t,\sigma )$.

### Proposition 1

*An instance* $((T;t,\sigma ), S)$*of the GTC problem admits a solution if and only if there exists a binary refinement* $S^*$*of* *S* *that agrees with and, therefore, displays* $\mathcal {R}(T;t,\sigma )$*such that* $A({T},{S^*})$*is acyclic.*

### Proof

Assume that $((T;t,\sigma ), S)$ admits a solution $\widehat{S}$. By Lemma 1, any binary refinement $S^*$ of $\widehat{S}$ displays $\mathcal {R}(T;t,\sigma )$ (and hence agrees with it) and $A({T},{S^*})$ is acyclic.

Conversely, suppose that there is a binary species tree $S^*$ that is a refinement of *S* and agrees with $\mathcal {R}(T;t,\sigma )$ such that $A({T},{S^*})$ is acyclic. Since $A({T},{S^*})$ is acyclic, we only need to show that $S^*$ displays $\mathcal {R}(T;t,\sigma )$. Let $ab|c \in \mathcal {R}(T;t,\sigma )$. Because $S^*$ is binary, we must have one of *ab*|*c*, *ac*|*b* or *bc*|*a* in $rt(S^*)$. Since $S^*$ agrees with $\mathcal {R}(T;t,\sigma )$, $ab|c \in rt(S^*)$, and it follows that $\mathcal {R}(T;t,\sigma )\subseteq rt(S^*)$. Hence, $S^*$ displays $\mathcal {R}(T;t,\sigma )$. Taking the latter arguments together, $S^*$ is a solution to the instance $((T;t,\sigma ), S)$ of the GTC problem, which completes the proof. $\square$

## An algorithm for the GTC problem

We need to introduce a few more concepts before describing our algorithm. For a sequence $Q=(v_1,\ldots ,v_k)$ we denote $\mathcal {M}(Q) = \{v_1,\ldots ,v_k\}$.

Given a graph *G*, a *partial topological sort* of *G* is a sequence of distinct vertices $Q = (v_1, v_2, \ldots , v_k)$ such that for each $i \in \{1,\dots ,k\}$, vertex $v_i$ has in-degree 0 in $G - \{v_1, \ldots , v_{i-1}\}$. If, for any $v \in V(G)$, there is no partial topological sort $Q'$ satisfying $\mathcal {M}(Q') = \mathcal {M}(Q) \cup \{v\}$ then *Q* is called a *maximal topological sort*. Note that the set of vertices in a maximal topological sort of *G* is unique, in the sense that for two distinct maximal topological sorts $Q,Q'$ of *G* we always have $\mathcal {M}(Q) = \mathcal {M}(Q')$ (cf. Lemma 4 in Appendix: "Properties of maximal topological sort and Lemma 4").

The motivation to consider partial and maximal topological sorts is as follows: To find a solution $S^*$ for a given GTC instance, we must ensure that the graph $A({T},{S^*})$ is acyclic which holds precisely when there is a maximal topological sort that contains every vertex (cf. Lemma 4 in Appendix: "Properties of maximal topological sort and Lemma 4"). If this is not the case, our goal is to find a refinement $S'$ of $S^*$ such that the updated graph $A({T},{S'})$ admits the same maximal topological sort as $A({T},{S^*})$ together with (at least one) additional vertex appended to it. We thus attempt to extend the maximal topological sorts in a step-wise manner until it contains every vertex of $A({T},{S^*})$, or until no refinement on $S^*$ allows us to augment it.

Our algorithm will make use of what we call a *good split refinement*. To this end, we provide first.

### Definition 7

(*Split refinement*)

Let *S* be an almost binary tree and let *x* be a cherry of *S*. We say that a refinement $S'$ of *S* is a *split refinement (of S at x)* if $S'$ can be obtained from *S* by partitioning the set $\mathrm {ch}(x)$ of children of *x* into two non-empty subsets $X_1, X_2=\mathrm {ch}(x)\setminus X_1$, and applying the extensions $(x, X_1)$ and then $(x, X_2)$.

In other words, we split the children set of *x* into non-empty subsets $X_1$ and $X_2$, and add a new parent vertex above each subset of size 2 or more and connect *x* with the newly created parent(s) or directly with $x'$ whenever $X_i=\{x'\}$.

We note that the two $(x, X_1)$ and $(x, X_2)$ extensions yield a valid refinement of *S* since the set $X_2$ is a strict subset of the children of *x* in $S_{x, X_1}$. Also observe that a split refinement transforms an almost binary tree into another almost binary tree that has one additional binary internal vertex.

### Definition 8

(*Good split refinement*) Let $((T;t,\sigma ), S)$ be a GTC instance. Let *Q* be a maximal topological sort of $A({T},{S})$, and let $S'$ be a split refinement of *S* at some cherry vertex *x*. Then $S'$ is a *good split refinement* if the two following conditions are satisfied:

- $$S'$$ agrees with $$\mathcal {R}(T;t,\sigma )$$;
- All the in-neighbors of *x* in $A({T},{S'})$ belong to $\mathcal {M}(Q)$.

The intuition behind a good split refinement is that it refines *S* by creating an additional binary vertex. Moreover, this refinement maintains agreement with $\mathcal {R}(T;t,\sigma )$ and, more importantly, creates a new vertex of in-degree 0 in the auxiliary graph that can be used to extend the current maximal topological sort. Ultimately, our goal is to repeat this procedure until *Q* contains every vertex, at which point we will have attained an acyclic graph.

**Fig. 2:**
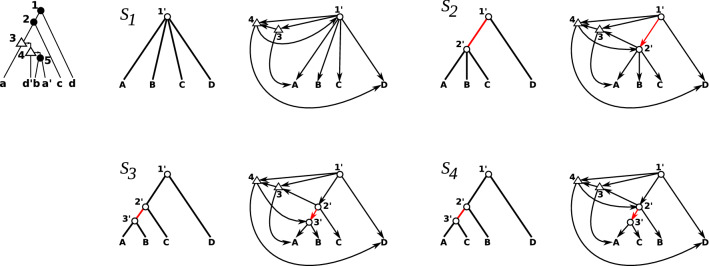
Top left: the gene tree $(T;t,\sigma )$ from Fig. 1 from which we obtain the species triplets $\mathcal {R}(T;t,\sigma ) = \{AB|D,AC|D\}$. Moreover, the sequence of species trees $S_1, S_2$ and $S_3$ is obtained by stepwise application of good split refinements. The species tree $S_4$ is an example of a split refinement of $S_2$ that is not good. The corresponding graphs $A({T},{S})$ are drawn right to the respective species tree *S*. For clarity, we have omitted to draw all vertices of $A({T},{S})$ that have degree 0. Moreover, in the respective species trees the planted roots are omitted. See text for further discussion

As an example consider Fig. 2. The species tree $S_1$ corresponds to the star tree. Clearly $S_1$ agrees with $\mathcal {R}(T;t,\sigma )$ since $rt(S_1)=\emptyset$. However, $A({T},{S})$ contains cycles. For the maximal topological sort $Q_1$ of $A({T},{S_1})$ we have $\mathcal {M}(Q_1) = L(T)\cup \{1,2,5\}$. Now, $S_2$ is a good split refinement of $S_1$, since $S_2$ agrees with $\mathcal {R}(T;t,\sigma )$ (in fact, $S_2$ displays $\mathcal {R}(T;t,\sigma )$) and since $x=1'$ has no in-neighbors in $A({T},{S_2})$ which trivially implies that all in neighbors of $x=1'$ in $A({T},{S_2})$ are already contained $\mathcal {M}(Q_1)$. For the maximal topological sort $Q_2$ of $A({T},{S_2})$ we have $\mathcal {M}(Q_2) = \mathcal {M}(Q_1)\cup \{1'\}$. Still, $A({T},{S_2})$ is not acyclic. The tree $S_3$ is a good split refinement of $S_2$, since $S_3$ agrees with $\mathcal {R}(T;t,\sigma )$ and the unique in-neighbor $1'$ of $x=2'$ in $A({T},{S_3})$ is already contained $\mathcal {M}(Q_2)$. Since $A({T},{S_3})$ is acyclic, there is a time-consistent reconciliation map from $(T;t,\sigma )$ to $S_3$, which is shown in Fig. 1. Furthermore, $S_4$ is not a good split refinement of $S_2$. Although $S_4$ is a split refinement of $S_2$ and agrees with $\mathcal {R}(T;t,\sigma )$, the in-neighbor 4 of $x=2'$ is not contained in $\mathcal {M}(Q_2)$.

We will discuss later the question of finding a good split refinement efficiently, if one exists. For now, assume that this can be done in polynomial time. The pseudocode for a high-level algorithm for solving the GTC problem is provided in Alg. 1. We note in passing that this algorithm serves mainly as a scaffold to provide the correctness proofs that are needed for the main Alg. 2.

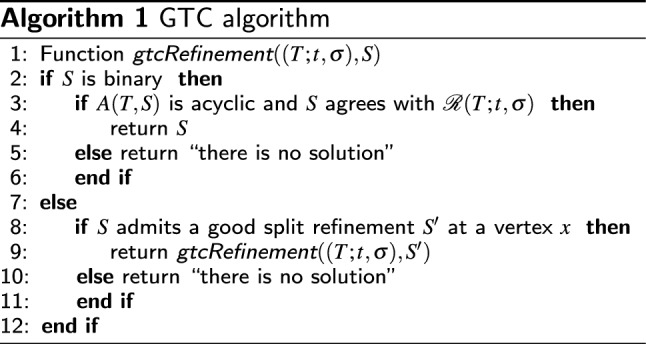



The following result shows that if we reach a situation where there is no good split refinement for an GTC instance, then no solution exits at all.

### Proposition 2

*Let* $((T;t,\sigma ), S)$*be a GTC instance such that* *S* *is not binary and does not admit a good split refinement. Then,* $((T;t,\sigma ), S)$*does not admit a solution.*

### Proof

See Appendix: "Proof of proposition". $\square$

We next show that if we are able to find a good split refinement $S'$ of *S*, the $((T; t, \sigma ), S')$ instance is equivalent in the sense that $((T; t, \sigma ), S)$ admits a solution if and only if $((T; t, \sigma ), S')$ also admits a solution.

### Theorem 3

*Let* $((T; t, \sigma ), S)$*be a GTC instance, and suppose that* *S* *admits a good split refinement* $S'$. *Then* $((T;t, \sigma ), S)$*admits a solution if and only if* $((T; t, \sigma ), S')$*admits a solution. Moreover, any solution for* $((T;t, \sigma ), S')$, *if any, is also a solution for* $((T;t,\sigma ), S)$.

### Proof

See Appendix: "Proof of Theorem". $\square$

### Theorem 4

*Algorithm* 1 *determines whether a given GTC instance* $((T; t, \sigma ), S)$*admits a solution or not and, in the affirmative case, constructs a solution* $S^*$ of $((T; t, \sigma ), S)$.

### Proof

Let $((T; t, \sigma ), S)$ be GTC instance. First it is tested in Line 2 whether *S* is binary or not. If *S* is binary, then *S* is already its binary refinement and Prop. 1 implies that *S* is a solution to $((T; t, \sigma ), S)$ if and only if *S* agrees with $\mathcal {R}(T;t,\sigma )$ and $A({T},{S})$ is acyclic. The latter is tested in Line 3. In accordance with Prop. 1, the tree *S* is returned whenever the latter conditions are satisfied and, otherwise, “there is no solution” is returned.

Assume that *S* is not binary. If *S* admits no good split refinement, then Alg. 1 (Line 10) returns “there is no solution”, which is in accordance with Prop. 2. Contrary, if *S* admits a good split refinement $S'$, then we can apply Theorem 3 to conclude that $((T; t, \sigma ), S)$ admits a solution if and only if $((T; t, \sigma ), S')$ admits a solution at all.

Now, we recurse on $((T; t, \sigma ), S')$ as new input of Alg. 1 in Line 8. The correctness of Alg. 1 is finally ensured by Theorem 3 which states that if $((T; t, \sigma ), S')$ admits a solution and thus, by Prop. 1, a binary refinement $S^*$ which is obtained by a series of good split refinements starting with *S*, is a solution for $((T; t, \sigma ), S)$. $\square$

### Finding a good split refinement

To find a good split refinement, if any, we can loop through each cherry *x* and ask “is there a good split refinement at *x*”? Clearly, every partition $X_1,X_2$ of $\mathrm {ch}(x)$ may provide a good split refinement and thus there might be $O(2^{|\mathrm {ch}(x)|})$ cases to be tested for each cherry *x*. To circumvent this issue, we define a second auxiliary graph that is an extension of the well-known Aho-graph to determine whether a set of triplets is compatible or not [47, 50, 51]. For a given set *R* of triplets, the Aho-graph has vertex set *V* and (undirected) edges *ab* for all triplets $ab|c\in R$ with $a,b,c\in V$. Essentially we will use this Aho-graph and add additional edges to it. The connected components of this extended graph eventually guide us to the process of finding good split refinements.

We define now the new auxiliary graph to determine whether the cherry *x* of *S* admits a good split refinement or not.

#### Definition 9

(*Good-split-graph*) Let $(T;t,\sigma )$ be a gene tree, *S* be a species tree and *x* be a cherry of *S*. Moreover, let *Q* be a maximal topological sort of $A({T},{S})$.

We define $G((T; t, \sigma ), S, x) = (V, E)$ as the undirected graph with vertex set $V = L(S(x))$. Moreover, an (undirected) edge *ab* is contained in *E* if and only if $a,b \in L(S(x))$ and *a*, *b* are distinct and satisfy at least one of the following conditions:

(C1): There exists $c \in L(S(x))$ such that $ab|c \in \mathcal {R}(T; t,\sigma )$;
(C2): There exists an edge $(u, v) \in E(T)$ such that $t(u) \in \{\mathfrak {d}, \mathfrak {t}\}$, $u \notin \mathcal {M}(Q)$, $t(v) = \mathfrak {s}$, and $\{a, b\} \subseteq \sigma _{T_{\mathcal {\overline{E}}}}(v)$;
(C3): There exists an edge $(u, v) \in E(T)$ such that $t(u) = t(v) = \mathfrak {s}$, $\widehat{\mu }_{S}(u) = x$ and $\{a, b\} \subseteq \sigma _{T_{\mathcal {\overline{E}}}}(v)$;
(C4): There exists a vertex $u \in V(T) \setminus \mathcal {M}(Q)$ such that $t(u) \in \{\mathfrak {d}, \mathfrak {t}\}$ and $\{a, b\} \subseteq \sigma _{T_{\mathcal {\overline{E}}}}(u)$.

Intuitively, edges represent pairs of species that must belong to the same part of a split refinement at *x*. That is, (C1) links species that would contradict a triplet of $\mathcal {R}(T; t, \sigma )$ if they were separated (as in the classical BUILD algorithm [47, 50, 51]); (C2) links species that would yield an A1-edge from a vertex not in *Q* into *x* if they were separated; (C3) links species that would create a self-loop on *x* if they were separated; and (C4) links species that would create an A3-edge from a vertex not in *Q* into *x* if separated. We want the graph $G((T; t, \sigma ), S, x)$ to be disconnected which would allow us to split the children of *x* while avoiding all the situations in which we create a separation of two children where we cannot ensure that this separation yields a good split refinement at *x*. Considering only such pairs of children turns out to be necessary and sufficient, and Theorem 5 below formalizes this idea.

#### Definition 10

Given an undirected graph *H*, we say that (*A*, *B*) is a *disconnected bipartition* of *H* if $A \cup B = V(H)$, $A \cap B = \emptyset$ and for each $a \in A, b \in B$, $ab \notin E(H)$.

We are now in the position to state how good split refinements can be identified. Note, we may assume w.l.o.g. that *S* agrees with $\mathcal {R}$, as otherwise there can be no good split refinement at all.

#### Theorem 5

*Let* $((T; t, \sigma ), S)$*be a GTC instance, and assume that* *S* *agrees with* $\mathcal {R}(T; t, \sigma )$. *Let* *Q* *be a maximal topological sort of* $A({T},{S})$. *Then there exists a good split refinement of* *S* *if and only if there exists a cherry x of S such that every strict ancestor of* *x* *in* *S* *is in* *Q*, *and such that* $G((T; t, \sigma ), S, x)$*is disconnected.*

*In particular, for any disconnected bipartition* (*A*, *B*) *of* *G*, *the split refinement that partitions the children of* *x* *into* *A* *and* *B* *is a good split refinement.*

#### Proof

See Appendix: "Proof of Theorem". $\square$

### The GTC algorithm

**Fig. 3:**
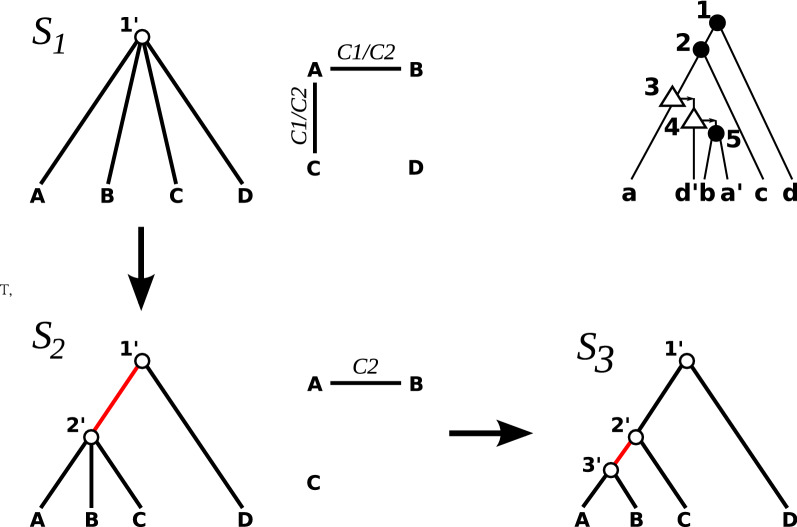
Top right: the gene tree $(T;t,\sigma )$ from Fig. 1 from which we obtain the species triplets $\mathcal {R}(T;t,\sigma ) = \{AB|D,AC|D\}$. We start with the star tree $S_1$ (top left) and obtain $G((T; t, \sigma ), S_1, 1')$, which is shown right to $S_1$. $G((T; t, \sigma ), S_1, 1')$ has four vertices *A*, *B*, *C*, *D* and two edges. The edge labels indicate which of the conditions in Def. 9 yield the respective edge. In $G((T; t, \sigma ), S_1, 1')$, there is only one non-trivial connected component which implies the good split that results in the tree $S_2$ (lower left). There is only one cherry $2'$ in $S_2$ and the corresponding graph $G((T; t, \sigma ), S_2, 2')$ is drawn right to $S_2$. Again, the connected components give a good split that results in the binary tree $S_3$. The tree $S_3$ is precisely the species tree (planted root omitted) as shown in the middle of Fig. 1

We can finally describe the detailed algorithm for the GTC problem, see also Fig. 3.

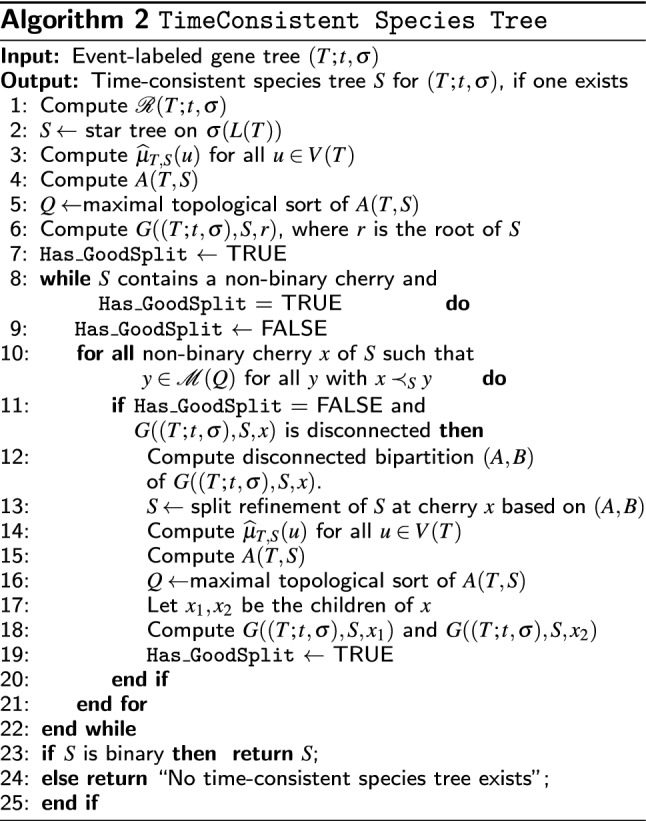



A pseudocode to compute a time-consistent species for a given event-labeled gene tree $(T;t,\sigma )$, if one exists, is provided in Alg. 2. The general idea of Alg. 2 is as follows. With $(T;t,\sigma )$ as input, we start with a star tree *S* and stepwisely refine *S* by searching for good split refinements. If in each step a good split refinement exists and *S* is binary (in which case we cannot further refine *S*), then we found a time-consistent species tree *S* for $(T;t,\sigma )$. In every other case, the algorithm returns “No time-consistent species tree exists”. Observe that at any point during the execution of the algorithm, the graph $G((T;t,\sigma ), S, x)$ is stored in memory for each cherry *x* of *S*. Since *S* is initialized as a star tree, only $G((T;t,\sigma ), S, r)$ needs to be computed initially, with *r* being the root of *S*. After applying a binary refinement at some non-binary cherry *x*, two cherries $x_1$ and $x_2$ are created, in which case we compute their auxiliary graph. Note that at this point, the graph $G((T;t,\sigma ), S, x)$ is not needed since *x* is not a cherry anymore, and thus it could be discarded. The correctness proof as well as further explanations on the running time are provided in the proof of Theorem 6.

To show that this algorithm runs in $O(n^3)$ time, we need first the following.

#### Lemma 2

$\mathcal {R}(T; t, \sigma )$*can be computed in worst-case time* $\Theta (n^3)$, *where* $n = |L(T)|$.

#### Proof

See Appendix: "Proof of Lemma". $\square$

We note that it is not too difficult to show that Algorithm 2 can be implemented to take time $O(n^4)$. Indeed, each line of the algorithm can be verified to take time $O(n^3)$, including the construction of $A({T},{S})$ (which takes time $O(n \log n)$, as shown in [4, Thm. 6]) and the construction of the $G((T;t,\sigma ), S, x)$ graphs (by checking every triplet of $\mathcal {R}(T; t, \sigma )$ for (C1) edges, and for every pair *a*, *b* of vertices, checking every member of $V(T) \cup E(T)$ for (C2), (C3) or (C4) edges). Since the main *while* loop is executed *O*(*n*) times, this yields complexity $O(n^4)$. However, with a little more work, this can be improved to cubic time algorithm.

As stated in Lemma 2, we may have $\mathcal {R}(T;t,\sigma ) \in \Theta (n^3)$. Thus, any hope of achieving a better running time would require a strategy to reconstruct a species tree *S* without reconstructing the full triplet set $\mathcal {R}(T;t,\sigma )$ that *S* needs to display. It may be possible that such an algorithm exists, however, this would be a quite surprising result and may require a completely different approach.

#### Theorem 6

*Algorithm* 2 *correctly computes a time-consistent binary species tree for* $(T;t,\sigma )$, *if one exists, and can be implemented to run in time* $O(n^3)$, *where* $n = |L(T)|$. *Its worst-case runtime is* $\Theta (n^3)$.

#### Proof

See Appendix: Proof of Theorem. $\square$

## Summary and outlook

Here, we considered event-labeled gene trees $(T;t,\sigma )$ that contain speciation, duplication and HGT. We solved the Gene Tree Consistency (GTC) problem, that is, we have shown how to decide whether a time-consistent species tree *S* for a given gene tree $(T;t,\sigma )$ exists and, in the affirmative case, how to construct such a binary species tree in cubic-time. Since our algorithm is based on the informative species triplets $\mathcal {R}(T;t,\sigma )$, for which $\mathcal {R}(T;t,\sigma )\in \Theta (n^3)$ may possible, there is no non-trivial way to improve the runtime of our algorithm. Our algorithm heavily relies on the structure of an auxiliary graph $A({T},{S})$ to ensure time-consistency and good split refinements to additionally ensure that the final tree *S* displays $\mathcal {R}(T;t,\sigma )$.

This approach may have further consequence in phylogenomics. Since event-labeled gene trees $(T;t,\sigma )$ can to some extent directly be inferred from genomic sequence data, our method allows to test whether $(T;t,\sigma )$ is “biologically feasible”, that is, there exists a time-consistent species tree for $(T;t,\sigma )$. Moreover, our method also shows that all information about the putative history of the species is entirely contained within the gene trees $(T;t,\sigma )$ and thus, in the underlying sequence data to obtain $(T;t,\sigma )$.

We note that the constructed binary species tree is one of possibly exponentially many other time-consistent species trees for $(T;t,\sigma )$. In particular, there are many different ways to choose a good split refinement, each choice may lead to a different species tree. Moreover, the reconstructed species trees here are binary. This condition might be relaxed and one may obtain further species tree by “contracting” edges so that the resulting non-binary tree is still a time-consistent species tree for $(T;t,\sigma )$. This eventually may yield so-called “least-resolved” time-consistent species trees and thus, trees that make no further assumption on the evolutionary history than actually supported by the data.

As part of further work, it may be of interest to understand in more detail, if our approach can be used to efficiently list all valid (possibly exponentially many) solutions, that is, all time-consistent species trees for $(T;t,\sigma )$.

## Figures and Tables

**Fig. 4:**
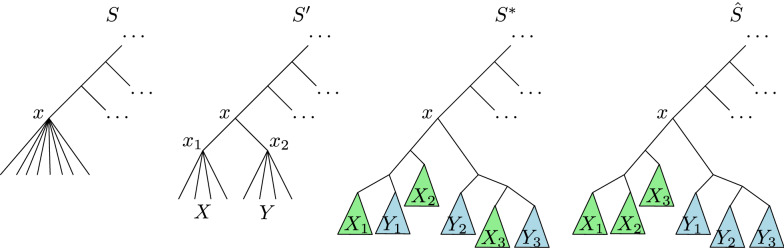
A representation of the trees $S, S', S^*$ and $\widehat{S}$. The $X_1, X_2$ and $X_3$ triangles represent subtrees containing only leaves from *X* (same with $Y_1, Y_2, Y_3$ and *Y*)

## Appendix A: Proof of results

### A.1 Lemma 3 and Proof of Lemma 1

Before we can prove Lemma 1, we provide a results that deals with possible self-loops in $A({T},{S})$. Although it may be possible to show in general (with a tedious case-by-case analysis) that a leaf *l* of *S* would never create a self-loop (*l*, *l*) in $A({T},{S})$ we use a conceptionally simpler idea and treat self-loops as a special case. In particular, we show that self-loops in $A({T},{S})$ cannot occur as long as there is a (not necessarily time-consistent) reconciliation map from $(T;t,\sigma )$ to *S*. In this case, the non-occurrence of self-loops is preserved by any graph $A({T},{S^*})$ for which $S^*$ is a refinement of *S*.

#### Lemma 3

*Let* $(T;t,\sigma )$*be an event-labeled gene tree,* *S* *be a species tree and* $S^*$*be a refinement of* *S*. *Moreover, let* *l* *be a leaf of* *S* (*and thus, of* $S^*$). *Then* (*l*, *l*) *is an edge of* $A({T},{S})$*if and only if* (*l*, *l*) *is an edge of* $A({T},{S^*})$.

*Furthermore, if there is a reconciliation map from* $(T;t,\sigma )$*to* *S* *then, the graph* $A({T},{S})$*will never contain self-loops and every edge* $(u',v')$*in* $A({T},{S})$*with* ${u',v'\in V(S)}$, *is either an A1- or A2-edge and satisfies* $v'\prec _S u'$.

#### Proof

Let $(T;t,\sigma )$ be an event-labeled gene tree, *S* be a species tree and $S^*$ be a refinement of *S*. Note that if (*l*, *l*) is a self-loop of $A({T},{S})$ (respectively $A({T},{S^*})$), then (*l*, *l*) must be an A1-edge, and so there is $(u, v) \in E(T)$ such that $\widehat{\mu }_S(u) = \widehat{\mu }_S(v) = l$ (respectively $\widehat{\mu }_{S^*}(u) = \widehat{\mu }_{S^*}(v) = l$). Since *l* is a leaf, $\widehat{\mu }_S(u) = \widehat{\mu }_S(v) = l$ if and only if $\widehat{\mu }_{S^*}(u) = \widehat{\mu }_{S^*}(v) = l$, and the statement follows.

For the second statement, assume that there is a reconciliation map from $(T;t,\sigma )$ to *S*. To see that $A({T},{S})$ does not contain self-loops, observe once again that self-loops can only be provided by A1-edges. So assume, for contradiction, that there is an edge $(u, v) \in E(T)$ such that $t(u),t(v)\in \{\odot , \mathfrak {s}\}$ and $\widehat{\mu }(u)=\widehat{\mu }(v)$. Since $t(u),t(v)\in \{\odot , \mathfrak {s}\}$, Property (M1) and (M2.i) imply that $\widehat{\mu }(u)=\mu (u)$ and $\widehat{\mu }(v)=\mu (v)$ for every reconciliation map $\mu$ from $(T;t,\sigma )$ to *S*. Since $v\prec _T u$, Condition (M3.ii) implies that $\widehat{\mu }(v) = \mu (v)\prec _S\mu (u)=\widehat{\mu }(u)$; a contradiction.

Now let $(u',v')$ be an edge in $A({T},{S})$ with $u',v'\in V(S)$. Since all A3- or A4-edges involve vertices of *T*, we can conclude that $(u',v')$ must either be an A1-edge or an A2-edge. Clearly, if $(u',v')$ is an A2-edge, we trivially have $v'\prec _S u'$. Assume that $(u',v')$ is an A1-edge. Hence there is an edge $(u,v)\in E(T)$ such that $u' = \widehat{\mu }(u)$ and $v' = \widehat{\mu }(v)$. This implies that $t(u),t(v)\in \{\mathfrak {s},\odot \}$. Condition (M1) and (M2.i) imply $\widehat{\mu }(u) = \mu (u)$ and $\widehat{\mu }(v) = \mu (v)$. Moreover, since $(u,v)\in E(T)$, we have $t(u)=\mathfrak {s}$. Now, we can apply Condition (M3.ii) to conclude that $v' = \widehat{\mu }(v)=\mu (v) \prec _S \mu (u) = \widehat{\mu }(u) = u'$. $\square$

We are now in the position to prove Lemma 1.

#### Proof of Lemma 1

We may assume that $\widehat{S}$ is non-binary as otherwise we are done. Let $S^*$ be any refinement of $\widehat{S}$. First observe that we have $\mathcal {R}(T;t,\sigma )\subseteq rt(\widehat{S}) \subseteq rt(S^*)$, and thus $S^*$ displays $\mathcal {R}(T;t,\sigma )$.

It remains to show that $A({T},{S^*})$ is acyclic. We first prove that any single $(x,X')$ extension applied to $\widehat{S}$ preserves acyclicity. Let $S'{:}\,{=}\,\widehat{S}_{x,X'}$ be any tree obtained from $\widehat{S}$ after applying some $(x,X')$ extension. As specified in Definition 5, if $|X'| \le 1$, then $S'=\widehat{S}$. In this case, $A({T},{S'}) = A({T},{\widehat{S}})$ is acyclic and we are done. Hence, suppose that $|X|>1$. Thus, a new node *y* was created, added as a child of *x* and became the new parent of $X' \subset X$. We claim that $A({T},{S'})$ is acyclic. For the remainder, we will write $\widehat{\mu }_{\widehat{S}}$ and $\widehat{\mu }_{S'}$ instead of $\widehat{\mu }_{T,\widehat{S}}$ and $\widehat{\mu }_{T, S'}$ since *T* will remain fixed. We will make use of the following properties.

1. For every subset $Z \subseteq L(S)$, it holds that $${\text {lca}}_{\widehat{S}}(Z) = {\left\{ \begin{array}{ll} {\text {lca}}_{S'}(Z) &{}\hbox { if}\ {\text {lca}}_{S'}(Z)\ne y \\ x &{}\text{ otherwise } \end{array}\right. }$$
2. For every $u\in V(T)$, it holds that $$\widehat{\mu }_{\widehat{S}}(u) = {\left\{ \begin{array}{ll} \widehat{\mu }_{S'}(u) &{}\text{ if } \widehat{\mu }_{S'}(u) \ne y \textit{(Case P2.a)} \\ x &{}\text{ otherwise } \textit{(Case P2.b)} \end{array}\right. }$$

Property (P1) follows from the fact that $L(S'(v)) = L(\widehat{S}(v))$ for any $v \in V(S') \setminus \{y\}$ and $L(S'(y)) \subset L(\widehat{S}(x))$. Therefore if ${\text {lca}}_{S'}(Z) = z\ne y$, then *z* is also a common ancestor of *Z* in $\widehat{S}$ and there cannot be lower common ancestor below *z*. If $z=y$, then *x* is a common ancestor of *Z* in $\widehat{S}$ and there cannot be a lower common ancestor below *x*. Property (P2) is a direct consequence of (P1) and the definition of $\widehat{\mu }_{S'}$ and $\widehat{\mu }_{\widehat{S}}.$

Now, suppose for contradiction that $A({T},{S'})$ contains a cycle $C = (w_1, \ldots , w_k, w_1)$. Note that $\mathcal {R}(T;t,\sigma )\subseteq rt(\widehat{S}) \subseteq rt(S')$. Thus, Theorem 1 implies that there is a reconciliation map from from $(T;t,\sigma )$ to $S'$. By Lemma 3, $A({T},{S'})$ does not contain self-loops and thus $k>1$ for $C = (w_1,\ldots , w_k, w_1)$

Consider the sequence of vertices ${\widetilde{C}} = ({\widetilde{w}}_1, \ldots , {\widetilde{w}}_k, {\widetilde{w}}_1)$ of vertices of $A({T},{\widehat{S}})$ where we take *C*, but replace *y* by *x* if it occurs. That is, we define, for each $1 \le i \le k$:

$$\begin{aligned} {\widetilde{w}}_i = {\left\{ \begin{array}{ll} w_i &{}\hbox { if}\ w_i \ne y\\ x &{}\hbox { if}\ w_i = y \end{array}\right. } \end{aligned}$$

We claim that every element in $\{({\widetilde{w}}_1, {\widetilde{w}}_2), \ldots , ({\widetilde{w}}_{k-1}, {\widetilde{w}}_k),({\widetilde{w}}_k, {\widetilde{w}}_1)\} \setminus \{(x, x)\}$ is an edge in $A({T},{\widehat{S}})$ (the pair (*x*, *x*) can occur in ${\widetilde{C}}$ if (*x*, *y*) is in *C*, but we may ignore it since this still results in a cycle). This will imply the existence of a cycle in $A({T},{\widehat{S}})$, yielding a contradiction.

We show that $({\widetilde{w}}_1, {\widetilde{w}}_2) \in E(A({T},{\widehat{S}}))$, assuming that $({\widetilde{w}}_1, {\widetilde{w}}_2) \ne (x, x)$. This is sufficient to prove our claim, since we can choose $w_1$ as any vertex of *C* and relabel the other vertices accordingly.

- *Case:* $(w_1, w_2)$*is an A1-edge.*
  Since $(w_1, w_2)$ is an A1-edge, it is defined by some edge $(u, v) \in E(T)$ and must coincide with one of the edges in ${\mathcal {A}} = \{(u, v), (u, \widehat{\mu }_{S'}(v)), (\widehat{\mu }_{S'}(u), v), (\widehat{\mu }_{S'}(u), \widehat{\mu }_{S'}(v))\}$.
  Suppose that $w_1,w_2\ne y$. Then, by construction of ${{\widetilde{w}}}_1$ and ${{\widetilde{w}}}_2$, we have ${{\widetilde{w}}}_1=w_1$ and ${{\widetilde{w}}}_2=w_2$. Hence, $({\widetilde{w}}_1, {\widetilde{w}}_2) = (w_1, w_2)$ is an edge in ${\mathcal {A}}$. By (P2), $\widehat{\mu }_{{\widehat{S}}}(u)=\widehat{\mu }_{S'}(u)$ and $\widehat{\mu }_{{\widehat{S}}}(v)=\widehat{\mu }_{S'}(v)$. Hence, $({\widetilde{w}}_1, {\widetilde{w}}_2)$ is of one of the form $(u, v), (u, \widehat{\mu }_{{\widehat{S}}}(v)), (\widehat{\mu }_{{\widehat{S}}}(u), v), (\widehat{\mu }_{{\widehat{S}}}(u), \widehat{\mu }_{{\widehat{S}}}(v))$. This implies that $({\widetilde{w}}_1, {\widetilde{w}}_2)$ is an A1-edge that is contained in $A({T},{\widehat{S}})$.
  If $w_1 =y$, then $y\in V(S')$ implies that $y=\widehat{\mu }_{S'}(u)$. By construction and (P2.b), ${\widetilde{w}}_1 = x = \widehat{\mu }_{{\widehat{S}}}(u)$. This, in particular, implies that $w_2 \notin \{x, y\}$ as otherwise, ${\widetilde{w}}_2 = x$; contradicting $({\widetilde{w}}_1, {\widetilde{w}}_2) \ne (x, x)$. By construction of ${{\widetilde{w}}}_2$, we have ${\widetilde{w}}_2=w_2$. Thus, $({\widetilde{w}}_1,{\widetilde{w}}_2)$ is either of the form $(\widehat{\mu }_{{\widehat{S}}}(u), v)$ or $(\widehat{\mu }_{{\widehat{S}}}(u), \widehat{\mu }_{{\widehat{S}}}(v))$ depending on the label *t*(*v*). In either case, $({\widetilde{w}}_1,{\widetilde{w}}_2)$ is an A1-edge that is contained in $A({T},{\widehat{S}})$ (invoking (P2.b) for the latter case).
  If $w_2 = y$ then, by analogous arguments as in the case $w_1=y$, we have ${\widetilde{w}}_2 = x = \widehat{\mu }_{{\widehat{S}}}(v)$ and ${\widetilde{w}}_1=w_1$. Again, $({\widetilde{w}}_1,{\widetilde{w}}_2)$ is an A1-edge that is contained in $A({T},{\widehat{S}})$.
  In summary, $({\widetilde{w}}_1,{\widetilde{w}}_2)$ is an A1-edge in $A({T},{\widehat{S}})$ whenever $(w_1, w_2)$ is an A1-edge in $A({T},{S'})$
- *Case:* $(w_1, w_2)$*is an A3-edge.*
  Since $(w_1, w_2)$ is an A3-edge, we have $(w_1, w_2) = (u, \widehat{\mu }_{S'}(u))$. Since $u \in V(T)$, it holds that $w_1=u \ne y$ and thus, ${\widetilde{w}}_1=w_1=u$. Now we can apply similar arguments as in the first case: either $\widehat{\mu }_{S'}(u) \ne y$ and thus, ${\widetilde{w}}_2 = w_2 = \widehat{\mu }_{S'}(u) = \widehat{\mu }_{\widehat{S}}(u)$ or $\widehat{\mu }_{S'}(u) =y$ and thus, ${\widetilde{w}}_2 = x = \widehat{\mu }_{\widehat{S}}(u)$. In both cases, $({\widetilde{w}}_1, {\widetilde{w}}_2) = (u,\widehat{\mu }_{\widehat{S}}(u))$ which implies that $({\widetilde{w}}_1, {\widetilde{w}}_2)$ is an A3-edge in $A({T},{S'})$.
- *Case:* $(w_1, w_2)$*is an A2-edge.*
  Since $(w_1, w_2)$ is an A2-edge, we have $(w_1, w_2)\in E(S')$ and hence, $w_1$ is the parent of $w_2$ in $S'$. This implies that $w_2 \ne y$ as, otherwise, $w_1=x$ and thus, $({\widetilde{w}}_1, {\widetilde{w}}_2) = (x, x)$; a contradiction. Thus, by construction, ${\widetilde{w}}_2 = w_2$. If $w_1 = y$, then ${{\widetilde{w}}}_1 = x$ and, by construction of $S'$, we have $(x, w_2) = ({\widetilde{w}}_1, {\widetilde{w}}_2) \in E(\widehat{S})$. In this case, $({\widetilde{w}}_1, {\widetilde{w}}_2)$ is an A2-edge in $E(A({T},{\widehat{S}}))$. Otherwise, if $w_1\ne y$, then ${{\widetilde{w}}}_1=w_1$. Hence, $(w_1, w_2) = ({\widetilde{w}}_1, {\widetilde{w}}_2) \in E(\widehat{S})$ which implies that $({\widetilde{w}}_1, {\widetilde{w}}_2)$ is an A2-edge in $E(A({T},{\widehat{S}}))$.
- *Case:* $(w_1, w_2)$*is an A4-edge.*
  Since $(w_1, w_2)$ is an A4-edge, there is an edge $(u,v)\in \mathcal {E}_T$ such that $w_1 = {\text {lca}}_{S'}(\widehat{\mu }_{S'}(u),\widehat{\mu }_{S'}(v))$ and $w_2=u$. Clearly, $w_2=y$ is not possible, since $w_2 = u$ corresponds to a vertex in *T*. By construction, ${{\widetilde{w}}}_2=w_2=u$. Note that in $A({T},{\widehat{S}})$, (*u*, *v*) defines the A4 edge $({\text {lca}}_{\widehat{S}}(\widehat{\mu }_{\widehat{S}}(u),\widehat{\mu }_{\widehat{S}}(v)), u)$. Therefore, it remains to show that ${{\widetilde{w}}}_1 = {\text {lca}}_{\widehat{S}}(\widehat{\mu }_{\widehat{S}}(u),\widehat{\mu }_{\widehat{S}}(v))$. Notice that
  $$\begin{aligned} w_1&= {\text {lca}}_{S'}(\widehat{\mu }_{S'}(u),\widehat{\mu }_{S'}(v)) \\&= {\text {lca}}_{S'}(lca_{S'}(\sigma _{T_{\mathcal {\overline{E}}}}(u)), lca_{S'}(\sigma _{T_{\mathcal {\overline{E}}}}(v))) \\&= {\text {lca}}_{S'}(\sigma _{T_{\mathcal {\overline{E}}}}(u) \cup \sigma _{T_{\mathcal {\overline{E}}}}(v)) \end{aligned}$$
  In a similar manner, we obtain
  $$\begin{aligned} {\text {lca}}_{\widehat{S}}(\widehat{\mu }_{\widehat{S}}(u), \widehat{\mu }_{\widehat{S}}(v)) = {\text {lca}}_{\widehat{S}}(\sigma _{T_{\mathcal {\overline{E}}}}(u) \cup \sigma _{T_{\mathcal {\overline{E}}}}(v)) \end{aligned}$$
  Let $Z = \sigma _{T_{\mathcal {\overline{E}}}}(u) \cup \sigma _{T_{\mathcal {\overline{E}}}}(v)$. Property (P1) implies that if $w_1 \ne y$, then ${\text {lca}}_{\widehat{S}}(\widehat{\mu }_{\widehat{S}}(u),\widehat{\mu }_{\widehat{S}}(v)) = {\text {lca}}_{\widehat{S}}(Z) = {\text {lca}}_{S'}(Z) = w_1 = {\widetilde{w}}_1$, as desired. If $w_1 = y$, then ${\text {lca}}_{\widehat{S}}(\widehat{\mu }_{\widehat{S}}(u),\widehat{\mu }_{\widehat{S}}(v)) = {\text {lca}}_{\widehat{S}}(Z) = x$ and ${\widetilde{w}}_1 = x$, as desired.

We have therefore shown that a cycle in $A({T},{S'})$ implies a cycle in $A({T},{\widehat{S}})$. Since $\widehat{S}$ is a solution, we deduce that $A({T},{S'})$ cannot have a cycle, and it is therefore also a solution to $((T;t,\sigma ), S)$.

To finish the proof, we need to show that $A({T},{S^*})$ is acyclic. This is now easy to see since $\widehat{S}$ can be transformed into $S^*$ by a sequence of extensions. As we showed, each extension maintains the acyclicity property, and we deduce that $A({T},{S^*})$ is acyclic. $\square$

### A.2 Properties of maximal topological sort and Lemma 4

#### Lemma 4

*Let* $G = (V,E)$*be a graph and* *Q* *and* $Q'$*be maximal topological sorts of* *G*. *Then,* $\mathcal {M}(Q) = \mathcal {M}(Q')$. *In particular,* $\mathcal {M}(Q) = V(G)$*if and only if* *G* *is a directed acyclic graph.*

*Furthermore, if* $x\in V\setminus \mathcal {M}(Q)$, *then none of the vertices* *y* *in* *V* *for which there is a directed path from* *x* *to* *y* *are contained in* $\mathcal {M}(Q)$.

*If* $x\in \mathcal {M}(Q)$, *then* *x* *is not contained in any cycle of* *G*.

#### Proof

Let $Q, Q'$ be maximal topological sorts of *G*, with $Q = (v_1, \ldots , v_k)$ and assume, for contradiction that $\mathcal {M}(Q) \ne \mathcal {M}(Q')$. Let $v_i$ be the first vertex in the sequence *Q* such that $v_i \notin \mathcal {M}(Q')$. Then all the in-neighbors of $v_i$ are in the set $\{v_1, \ldots , v_{i-1}\}$. Moreover, by assumption $\{v_1, \ldots , v_{i-1}\} \subseteq \mathcal {M}(Q')$, implying that $v_i$ has in-degree 0 in $G - \mathcal {M}(Q')$. Hence, we could append $v_i$ to $Q'$, contradicting its maximality. The fact that $\mathcal {M}(Q) = V(G)$ if and only if *G* a directed acyclic graph is well-known and follows from the results of [52].

Let $x\in V\setminus \mathcal {M}(Q)$. Moreover, let $P=(x=v_1,\dots v_k=y)$, $k\ge 2$, be a directed path from *x* to *y*. Since $x\notin \mathcal {M}(Q)$, $v_2$ has in-degree greater than 0 in $G-\mathcal {M}(Q)$. Therefore, $v_2\notin \mathcal {M}(Q)$ and, by induction, $v_k=y\notin \mathcal {M}(Q).$

We now show that no vertex $x\in \mathcal {M}(Q)$ can be contained in a cycle of *G*. Assume, for contradiction, that there is a cycle *C* such that some of its vertices are part of a maximal topological sort $Q = (v_1,\dots ,v_k)$ of *G*. Let $v_i$ be the first vertex of *C* that appears in *Q*. Hence, $v_i$ must have in-degree 0 in $G - \{v_1, \ldots , v_{i-1}\}$. But this implies, that the in-neighbor of $v_i$ in *C* must already be contained in *Q*; a contradiction. $\square$

A maximal topological sort of *G* can be found by applying the following procedure: start with *Q* empty, and while there is a vertex of in-degree 0 in $G - \mathcal {M}(Q)$, append *v* to *Q* and repeat. Then, *G* is acyclic if an only if any maximal topological sort *Q* of *V*(*G*) satisfies $\mathcal {M}(Q) = V(G)$. The latter argument is correct as it directly mirrors the well-known algorithm by Kahn to find a topological sort of graph [52].

### A.3 Proof of proposition 2

We prove some general-purpose statements first. Let $I_G(v)$ denote the set of in-neighbors of vertex *v* in a graph *G*.

#### Lemma 5

*Let* $((T;t,\sigma ), S)$*be a GTC instance. Moreover, let* $S'$*be a split refinement of* *S* *at a cherry* *x*. *Then, for every vertex* *y* *of* *S* *such that* $y \not \preceq _S x$, *it holds that* $I_{A({T},{S'})}(y) = I_{A({T},{S})}(y)$.

#### Proof

Let $y\in V(S)$ be a vertex of *S* satisfying $y \not \preceq _S x$. Since $y\notin V(T)$, for every $z\in I_{A({T},{S'})}(y)$ or $z\in I_{A({T},{S})}(y)$ the edge (*z*, *y*) cannot be an A4-edge.

If (*z*, *y*) is an A2-edge in $A({T},{S})$ then, $(z,y)\in E(S)$, which is if and only if $(z,y)\in E(S')$, since $y\not \preceq _S x$. In this case, $z\in I_{A({T},{S})}(y)$ if and only if $z\in I_{A({T},{S'})}(y),$

It remains to consider A1- and A3-edges. We translate here Property (P2.a) as in the proof of Lemma 1. It states as a particular case that for $u \in V(T)$, $\widehat{\mu }_{S}(u)=\widehat{\mu }_{S'}(u)$ whenever $\widehat{\mu }_{S}(u) \not \preceq x$. This holds for every $y \not \preceq _S x$, which immediately implies that $z\in I_{A({T},{S})}(y)$ and the edge (*z*, *y*) is an A1-edge, resp., A3-edge in $A({T},{S})$ if and only if $z\in I_{A({T},{S'})}(y)$ and (*z*, *y*) is an A1-edge, resp., A3-edge in $A({T},{S'})$. $\square$

We are now in the position to prove Prop.  2.

#### Proof of Prop. 2

We show that if *S* does not admit a good split refinement, then none of the binary refinements $S^*$ of *S* is a solution to the GTC instance $((T;t,\sigma ), S)$. Contraposition of Lemma 1 together with Prop. 1 then implies that there is no solution at all for $((T;t,\sigma ), S).$

Thus, assume that *S* is not binary (but almost binary, due to the definition of GTC instances) such that *S* does not admit a good split refinement. Let $S^*$ be any binary refinement of *S*. We may assume that $S^*$ agrees with and thus, displays $\mathcal {R}(T,t,\sigma )$, as otherwise it is not a solution. We show that $A({T},{S^*})$ contains a cycle.

Let *Q* be a maximal topological sort of $A({T},{S})$. By Lemma 4, $\mathcal {M}(Q)$ is independent of the choice of the particular sequence *Q*. Note that $V(S) \subseteq V(S^*)$ and therefore that $V(A({T},{S})) \subseteq V(A({T},{S^*})).$ In particular, $\mathcal {M}(Q) \subseteq V(A({T},{S^*}))$. Also notice that because of the A2 edges in $A({T},{S})$ and Lemma 4, if a vertex $x \in V(S)$ is not in $\mathcal {M}(Q)$, then no descendant of *x* in *S* is in $\mathcal {M}(Q).$ We separate the proof into three claims.

Claim 1 *Let* $y \in V(S^*) \setminus V(S)$. *Then* *y* *has in-degree at least* 1 *in* $A({T},{S^*})- \mathcal {M}(Q)$.

Observe that since $y \notin V(S)$, *y* must have been created by one of the extensions that transforms *S* into $S^*$, and so in $S^*$, *y* must be a descendant of a vertex *x* such that *x* is a non-binary cherry in *S*. We first argue that $x \notin \mathcal {M}(Q)$, and then proceed to prove the claim.

Note that since *x* is non-binary and $S^*$ is a binary refinement of *S*, there is a split refinement $S'$ of *S* at *x* such that $S^*$ refines $S'$. Since $S^*$ agrees with $\mathcal {R}(T;t,\sigma )$, also $S'$ agrees with $\mathcal {R}(T;t,\sigma )$. If all in-neighbors of *x* in $A({T},{S'})$ are in *Q*, then $S'$ is a good split refinement; a contradiction. So we may assume that *x* has an in-neighbor *y* in $A({T},{S'})$ such that $y \notin \mathcal {M}(Q)$. Since $x\in V(S)$, the edge (*y*, *x*) cannot be an A4-edge in $A({T},{S'})$. If (*y*, *x*) is an A1-edge in $A({T},{S'})$, then $x = \widehat{\mu }_{S'}(v)$ for some $v \in V(T)$. By construction, $L(S(x)) = L(S'(x))$ and thus, $\widehat{\mu }_{S}(v) = \widehat{\mu }_{S'}(v) = x$. Therefore, $(y, \widehat{\mu }_S(v)) = (y, x) \in E(A({T},{S}))$. Similarly, if (*y*, *x*) is an A3-edge in $A({T},{S'})$, then $x = \widehat{\mu }_{S'}(y)$ and again, $(y, \widehat{\mu }_S(y)) = (y, x) \in E(A({T},{S}))$. If (*y*, *x*) is an A2-edge in $A({T},{S'})$, then $(y, x) \in E(A({T},{S}))$ since the parent of *x* is the same in *S* and $S'$. In all cases, *y* is an in-neighbor of *x* in $A({T},{S})$. However, since $y \notin \mathcal {M}(Q)$, vertex *y* remains an in-neighbor of *x* in the graph $A({T},{S}) - \mathcal {M}(Q)$. It follows that $x \notin \mathcal {M}(Q)$.

Now, since $x \notin \mathcal {M}(Q)$, and because of the A2-edges, *y* must have in-degree at least 1 in $A({T},{S^*}) - \mathcal {M}(Q)$.

Claim 2 *Let* $v \in V(T) \setminus \mathcal {M}(Q)$. *Then* *v* *has in-degree at least* 1 *in* $A({T},{S^*}) - \mathcal {M}(Q)$.

Let $v \in V(T) \setminus \mathcal {M}(Q)$. Since $v \notin \mathcal {M}(Q)$, *v* has in-degree at least 1 in $A({T},{S}) - \mathcal {M}(Q)$, or else it could be added to the maximal topological sort. Let (*x*, *v*) be an incoming edge of *v* in $A({T},{S}) - \mathcal {M}(Q)$, which is either an A1- or an A4- edge.

If (*x*, *v*) is an A1- edge, we either have $x \in V(T)$ or $x \in V(S)$. Suppose first that $x \in V(T)$. In this case, the (*x*, *v*) edge exists because *x* is the parent of *v* in *T* with *t*(*x*), *t*(*v*) both in $\{\mathfrak {d}, \mathfrak {t}\}$. This is independent of *S*, and so (*x*, *v*) is also an A1-edge of $A({T},{S^*}) - \mathcal {M}(Q)$. Suppose now that $x \in V(S)$. In this case, observe that $x\notin \mathcal {M}(Q)$, since (*x*, *v*) is an edge in $A({T},{S}) - \mathcal {M}(Q)$. Therefore, $x \in V(S) \setminus \mathcal {M}(Q)$. This, in particular, implies that the parent $v_p$ of *v* in *T* satisfies $t(v_p) = \mathfrak {s}$ and $\widehat{\mu }_{S}(v_p) = x$. Since $S^*$ refines *S*, we must have $\widehat{\mu }_{S^*}(v_p) \preceq _{S^*} x$. There are two cases, either $\widehat{\mu }_{S^*}(v_p)\notin V(S)$, in which case trivially $\widehat{\mu }_{S^*}(v_p) \notin \mathcal {M}(Q)$, or $\widehat{\mu }_{S^*}(v_p)\in V(S)$. In the latter case, there is a directed (possibly edge-less) path from *x* to $\widehat{\mu }_{S^*}(v_p)$ in $A({T},{S})$ due to the A2-edges. Thus, we can apply Lemma 4 to conclude that $\widehat{\mu }_{S^*}(v_p) \notin \mathcal {M}(Q)$.

In either case, $(\widehat{\mu }_{S^*}(v_p), v)$ is an A1-edge of $A({T},{S^*}) - \mathcal {M}(Q)$. Therefore, *v* has an in-neighbor in $A({T},{S^*})$ that does not belong to *Q*.

Assume now that (*x*, *v*) is an A4-edge. Thus, there is an edge $(v,v') \in \mathcal {E}_T$ such that $x= lca_{S}(\widehat{\mu }_S(v), \widehat{\mu }_S(v'))$. Again since $S^*$ refines *S*, it is not hard to see that ${\text {lca}}_{S^*}(\widehat{\mu }_S^*(v), \widehat{\mu }_S^*(v')) \preceq _{S^*} x$. By similar arguments as before, ${\text {lca}}_{S^*}(\widehat{\mu }_S^*(v), \widehat{\mu }_S^*(v')) \notin \mathcal {M}(Q)$. Thus, $({\text {lca}}_{S^*}(\widehat{\mu }_S^*(v), \widehat{\mu }_S^*(v')),v)$ is an A4-edge of $A({T},{S^*})$. Hence, *v* also has in-degree at least 1 in $A({T},{S^*}) - \mathcal {M}(Q)$, which proves Claim 2.

We prove the analogous statement for the species tree vertices.

Claim 3 *Let* $x \in V(S) \setminus \mathcal {M}(Q)$. *Then* *x* *has in-degree at least* 1 *in* $A({T},{S^*}) - \mathcal {M}(Q)$.

Let $x \in V(S) \setminus \mathcal {M}(Q)$. We may assume that *x* has in-degree at least 1 in $A({T},{S}) - \mathcal {M}(Q)$, by the maximality of *Q*. Notice that since $S^*$ is a binary refinement of *S*, there exists a sequence of split refinements that transforms *S* into $S^*$. That is, there is a sequence of trees $S = S_1, S_2, \ldots , S_k = S^*$ such that for $2 \le i \le k$, $S_i$ is a split refinement of $S_{i-1}$.

Let (*w*, *x*) be an incoming edge of *x* in $A({T},{S}) - \mathcal {M}(Q)$. We consider the following three exclusive cases: either *x* is a binary or a non-binary interior vertex, or a leaf.

Suppose first that *x* is a binary vertex of *S*. Because *S* is almost binary, *x* is not a descendant of any non-binary vertex of *S*. By applying Lemma 5 successively on each split refinement of the sequence transforming *S* into $S^*$, we obtain $I_{A({T},{S_1})}(x) = I_{A({T},{S_2})}(x) = \ldots = I_{A({T},{S_k})}(x) = I_{A({T},{S^*})}(x)$. In particular, $w \in I_{A({T},{S^*})}(x)$, which proves the claim for this case since $w \notin \mathcal {M}(Q)$.

Suppose now that *x* is a leaf of *S*. If the parent $x_p$ of *x* is binary, then again, successive application of Lemma 5 on $S_1, \ldots , S_k$ implies that $I_{A({T},{S})}(x) = I_{A({T},{S^*})}(x)$, and therefore that $w \in I_{A({T},{S^*})}(x)$. If $x_p$ is a non-binary cherry, then $x_p \notin \mathcal {M}(Q)$ by Claim 1. There are two cases, either the parent *p*(*x*) of *x* in $S^*$ is identical to $x_p$ or not. In the first case, $p(x)=x_p$ is not part of *Q*. In the latter case, *p*(*x*) refers to some newly added vertex during the construction of $S^*$. In this case, *p*(*x*) is not contained in *S* and so neither in *Q*. In summary, the parent of *x* in $S^*$ is not in *Q*. Due to the A2-edges, *x* has in-degree at least 1 in $A({T},{S^*}) - \mathcal {M}(Q)$.

Finally, suppose that *x* is a non-binary interior vertex of *S*, i.e. *x* is a cherry. Let $S'$ be a split refinement of *S* at *x* such that $S^*$ refines $S'$. Recall that as in Claim 1, $S'$ agrees with $\mathcal {R}(T;t,\sigma )$. This and the fact that *S* does not admit a good split refinement implies that *x* has in-degree at least 1 in $A({T},{S'}) - \mathcal {M}(Q)$. Now, *x* is binary in $S'$. As before, there is a sequence of binary refinements transforming $S'$ into $S^*$. Since *x* is not a descendant of any non-binary vertex in $S'$, by applying Lemma 5 on each successive refinement, $I_{A({T},{S'})}(x) = I_{A({T},{S^*})}(x)$. It follows that *x* has in-degree at least 1 in $A({T},{S^*}) - \mathcal {M}(Q)$ as well. This proves Claim 3.

To finish the argument, note that $V(A({T},{S^*}) - \mathcal {M}(Q)) = (V(T) \setminus \mathcal {M}(Q)) \cup (V(S) \setminus \mathcal {M}(Q)) \cup (V(S^*) \setminus V(S))$. By Claim 1, each vertex in $V(S^*) \setminus V(S)$ has in-degree at least 1 in $A({T},{S^*}) - \mathcal {M}(Q)$, and by Claim 2 and Claim 3, it follows that every vertex of $A({T},{S^*}) - \mathcal {M}(Q)$ has in-degree at least 1. This implies that $A({T},{S^*}) - \mathcal {M}(Q)$ contains a cycle, and hence that $A({T},{S^*})$ also contains a cycle. We have reached a contradiction, proving the lemma. $\square$

### A.4 Proof of Theorem 3

First, we provide the following lemma for later reference.

#### Lemma 6

*Let* $((T;t,\sigma ), S)$*be a GTC instance and let* *Q* *be a maximal topological sort of* $A({T},{S})$. *Moreover, let* $S'$*be a split refinement of* *S* *at a cherry* *x*. *Then, for any maximal topological sort* $Q'$*of* $A({T},{S'})$, *it holds that* $\mathcal {M}(Q) \subseteq \mathcal {M}(Q')$.

#### Proof

Assume without loss of generality that the cherry *x* is non-binary in *S*, as otherwise $S=S'$ and we are done. Let $x_1, x_2$ be the children of *x* in $S'$, and assume furthermore w.l.o.g. that $|L(S'(x_1))| \ge |L(S'(x_2))|$. Note that $x_2$ could be a leaf, but that $x_1$ must be an internal vertex since *x* is a non-binary cherry. Now, if $\mathcal {M}(Q)=\emptyset$, then the lemma statement is trivially satisfied. Hence, assume that $Q = (w_1, \ldots , w_l)$, $l\ge 1$. We construct partial topological sorts $Q_0, Q_1, \ldots , Q_l$ of $A({T},{S'})$ as follows. Define $Q_0 = ()$ as an empty sequence and, for each $1 \le i \le l$, $Q_i$ is obtained from $Q_{i-1}$ by appending $w_i$ to $Q_{i-1}$ if $w_i \ne x$, and if $w_i = x$, by appending *x* and $x_1$ (in this order) to $Q_{i-1}$, and then appending $x_2$ to $Q_{i-1}$ if it is not a leaf in $S'$. We show, by induction, that each $Q_i$ is a partial topological sort of $A({T},{S'})$. The base case $i = 0$ is clearly satisfied. So let us assume that for $i > 0$ the sequence $Q_{i - 1}$ is a partial topological of of $A({T},{S'})$. Consider now the vertex $w_i$.

- *Case:* $$w_i \in V(S)$$*and* $$w_i \not \preceq _S x$$
  By Lemma 5, $I_{A({T},{S})}(w_i) = I_{A({T},{S'})}(w_i)$. Since each member of $I_{A({T},{S})}(w_i)$ precedes $w_i$ in *Q*, $\mathcal {M}(Q_{i-1})$ contains $I_{A({T},{S})}(w_i)$. It follows that appending $w_i$ to $Q_{i-1}$ yields a partial topological sort $Q_i$ of $A({T},{S'})$.

In the remaining cases, we will make frequent use of the fact that if $Q'$ is a partial topological sort of $A({T},{S'})$ and *v* is a vertex with $I_{A({T},{S'})}(v)\setminus M = \emptyset$ for some (possibly empty) subset $M\subseteq \mathcal {M}(Q')$, then appending *v* to $Q'$ yields a partial topological sort of $A({T},{S'})$. In other words, we can w.l.o.g. assume $I_{A({T},{S'})}(v)\setminus M\ne \emptyset$ for all such considered sets.

- *Case:* $$w_i \in V(S)$$*and* $$w_i = x$$
  We start by showing that the sequence $Q^x_{i-1}$ obtained by appending *x* to $Q_{i-1}$ is a partial topological sort of $A({T},{S'})$. Let $z \in I_{A({T},{S'})}(x)$. Suppose first that $z \in V(S')$. Then (*z*, *x*) is either an A1- or A2-edge of $A({T},{S'})$. If (*z*, *x*) is an A2-edge, then *z* is the parent of *x* in both *S* and $S'$. Thus $(z, x) \in E(A({T},{S}))$ and since $x \in \mathcal {M}(Q)$, we must have $z \in \mathcal {M}(Q)$. Moreover, *z* must precede *x* in *Q*, and it follows that $z \in \mathcal {M}(Q_{i-1})$. If (*z*, *x*) is an A1-edge, then $x \preceq _{S'} z$. If $x \prec _{S'} z$, then $x \prec _{S} z$ as well. Thus in $A({T},{S})$, there is a path of A2-edges from *z* to *x*, implying that *z* precedes *x* in *Q*. Finally if $x = z$, then $A({T},{S'})$ contains the self-loop (*x*, *x*). In this case, there is an edge $(u, v) \in E(T)$ such that $(x, x) = (\widehat{\mu }_{S'}(u), \widehat{\mu }_{S'}(v))$. By construction, $L(S'(x)) = L(S(x))$ and therefore, $(\widehat{\mu }_S(u), \widehat{\mu }_S(v)) = (x, x)$ is an edge of $A({T},{S})$. This case cannot occur, since it is impossible that $x \in \mathcal {M}(Q)$ if *x* is part of a self-loop. Therefore, *z* precedes *x* in *Q* whenever $z \in V(S')$.
  If instead $z \in V(T)$, then (*z*, *x*) is either an A1- or A3-edge of $A({T},{S'})$, in which case there is $z'\in V(T)$ such that $(z, x) = (z, \widehat{\mu }_{S'}(z'))$. By construction $L(S'(x)) =L(S(x))$ and therefore, $(z, \widehat{\mu }_{S}(z')) = (z, x)$ is an edge of $A({T},{S})$. Again, *z* must precede *x* in *Q*. We have thus shown that *z* precedes *x* in *Q* for every $z \in I_{A({T},{S'})}(x) \subseteq \mathcal {M}(Q_{i-1})$. Hence, appending *x* to $Q_{i-1}$ yields a partial topological sort $Q^x_{i-1}$ of $A({T},{S'})$.
  We continue with showing that $Q_{i}$ is a partial topological sort of $A({T},{S'})$. Note, $Q_{i}$ is obtained by appending $x_1$ and, in case $x_2$ is not a leaf in $S'$, also $x_2$ to the partial topological sort $Q^x_{i-1}$ of $A({T},{S'})$. Let $z \in I_{A({T},{S'})}(x_j)\setminus \{x\}$, where $x_j \in \{x_1, x_2\}$ is is chosen to be an interior vertex of $S'$. Note, $x_j=x_1$ is always possible as argued at the beginning of this proof. Suppose that $z \in V(S')$. In this case, $(z, x_j)$ cannot be an A2-edge since it would imply $x=z$; a contradiction. Hence, $(z, x_j)$ is an A1-edge of $A({T},{S'})$ and $x_j \preceq _{S'} z$. Similarly as before, if $x_j \prec _{S'} z$, then $x \prec _{S} z$ since $z \ne x$. Thus, *z* precedes *x* in *Q*, since $A({T},{S})$ contains a path of A2-edges from *z* to *x*. If $x_j = z$, then there is an edge $(u, v) \in E(T)$ such that $(\widehat{\mu }_{S'}(u), \widehat{\mu }_{S'}(v)) = (x_j, x_j)$. Since $x_j$ is supposed not to be a leaf in $S'$ and by construction of $S'$ from *S*, we must have in *S* that $(\widehat{\mu }_S(u), \widehat{\mu }_S(v)) = (x, x)$, contradicting $x \in \mathcal {M}(Q)$. Now, assume that $z \in V(T)$ in which case $(z, x_j)$ is either an A1- or A3-edge in $A({T},{S'})$. Again, there must be a vertex $z'\in V(T)$ such that $(z, x_j) = (z, \widehat{\mu }_{S'}(z'))$. By construction $L(S'(x_i)) \subset L(S(x))$. This and $x_j = \widehat{\mu }_{S'}(z')$ immediately implies that $x = \widehat{\mu }_{S}(z')$. Thus, $(z, \widehat{\mu }_{S}(z')) = (z, x)$ is an edge of $A({T},{S})$ and *z* must precede *x* in *Q*. Again, this holds for every *z* which implies implies that $I_{A({T},{S'})}(x_j) \setminus \{x\} \subseteq \mathcal {M}(Q^x_{i-1})$. Thus, appending $x_1$ and $x_2$ to $Q^x_{i-1}$ after *x* yields the partial topological sort $Q_i$ of $A({T},{S'})$.
- *Case:* $$w_i \in V(S)$$*and* $$w_i \prec _S x$$
  Since *x* is a cherry, $w_i$ must be a leaf in *S*. Thus *x* precedes $w_i$ in *Q* and therefore, we may assume that $x, x_1$ and, in case $x_2$ is not a leaf in $S'$, also $x_2$ are contained in the partial topological sort $Q_{i-1}$ of $A({T},{S'})$ Note, $x_2$ could be absent from $Q_{i-1}$ if it is a leaf and $w_i = x_2$. That is, we may assume that $w_i$ is a child of either $x, x_1$ or $x_2$ in $S'$, and that the parent of $w_i$ in $S'$ is in $Q_{i-1}$. Consider $z \in I_{A({T},{S'})}(w_i) \setminus \{x, x_1, x_2\}$. If $z \in V(S')$, then $w_i \preceq _{S'} z$. As before, if $w_i \prec _{S'} z$, then $w_i \prec _{S} z$ and because of the A2-edges of $A({T},{S})$, *z* precedes $w_i$ in *Q*. If $z = w_i$, then $(w_i, w_i)$ is a self-loop in $A({T},{S'})$. By Lemma 3, $(w_i, w_i)$ is also a self-loop of $A({T},{S})$; a contradiction since, by Remark 5, $A({T},{S})$ has no self-loops on its leaves. So assume that $z \in V(T)$. Then $(z, w_i)$ is an A1- or A3-edge. Since $w_i$ is a leaf, we have that for any $v \in V(T)$, $\widehat{\mu }_{S'}(v) = w_i$ if and only if $\widehat{\mu }_S(v) = w_i$. It follows that $(z, w_i) \in E(A({T},{S}))$. Therefore, *z* precedes $w_i$ in *Q* and *z* belongs to $Q_{i-1}$. Thus we may append $w_i$ to $Q_{i-1}$ to obtain a partial topological sort $Q_i$ of $A({T},{S'})$.
- *Case:* $$w_i \in V(T)$$
  Let $z \in I_{A({T},{S'})}(w_i)$. Thus, $(z,w_i)$ is either an A1- or A4-edge in $A({T},{S'})$. If $z \in V(T)$, then $(z,w_i)$ is an A1-edge in $A({T},{S'})$. Since the event-labels in *T* are fixed, $(z,w_i)$ is an A1-edge in $A({T},{S})$ and thus, $z \in I_{A({T},{S})}(w_i)$. Therefore, *z* precedes $w_i$ in *Q*.
  Now, suppose $z \in V(S')$. If $(z, w_i)$ is an A1-edge, then the parent *u* of $w_i$ in *T* satisfies $\widehat{\mu }_{S'}(u) = z$. If $z \notin \{x, x_1, x_2\}$, then we immediately obtain $\widehat{\mu }_{S}(u) = z$. Hence, $(z, w_i) \in E(A({T},{S}))$ and thus, *z* precedes $w_i$ in *Q*. If $z \in \{x, x_1, x_2\}$, then it is easy to verify that $\widehat{\mu }_S(u) = x$. Thus $(x, w_i) \in E(A({T},{S}))$, *x* precedes $w_i$ in *Q*. By construction, we have added $x,x_1,x_2$ in one of the previous steps to obtain $Q_j$, $1\le j\le i-1$. Hence, $z \in \{x, x_1, x_2\}$ precedes $w_i$ in *Q*. If instead $(z, w_i)$ is an A4-edge, then $w_i$ has a child *v* such that ${\text {lca}}_{S'}(\widehat{\mu }_{S'}(w_i), \widehat{\mu }_{S'}(v)) = z$. Clearly, it holds that $z = {\text {lca}}_{S'}(Z)$ for $Z = \sigma _{T_{\mathcal {\overline{E}}}}(w_i) \cup \sigma _{T_{\mathcal {\overline{E}}}}(v)$. If $z \in \{x, x_1, x_2\}$, then $({\text {lca}}_S(Z), w_i) = (x, v) \in E(A({T},{S}))$, and if $z \notin \{x, x_1, x_2\}$, then $({\text {lca}}_S(Z), w_i) = ({\text {lca}}_{S'}(Z), w_i) = (z, w_i) \in E(A({T},{S}))$. In both cases, *z* precedes $w_i$ in *Q*. In every case, each *z* is already contained in $Q_{i-1}$, and we may append $w_i$ to $Q_{i-1}$ to obtain a partial topological sort $Q_i$ of $A({T},{S'})$.

We have shown that $Q_l$ is a partial topological sort of $A({T},{S'})$ satisfying $\mathcal {M}(Q) \subseteq \mathcal {M}(Q_l)$. If we add in-degree 0 vertices in $Q_l$ until we obtain a maximal topological sort $Q'$ of $A({T},{S'})$, then have $\mathcal {M}(Q) \subseteq \mathcal {M}(Q_l) \subseteq \mathcal {M}(Q')$, as desired. $\square$

We are now in the position to prove Theorem 3.

#### Proof of Theorem 3

It is easy to see that any solution $S^*$ of $((T; t, \sigma ), S')$ would be a solution for $((T; t, \sigma ), S)$. Hence, if $((T; t, \sigma ), S)$ does not admit a solution, then $((T; t, \sigma ), S')$ cannot admit a solution.

For the converse, suppose now that $((T; t, \sigma ), S)$ admits a solution. Thus, there is a binary refinement $S^*$ of *S* that displays $\mathcal {R}(T;t,\sigma )$ and such that $A({T},{S^*})$ is acyclic. Let $S'$ be any good split refinement of *S* at some cherry *x* of *S*. Furthermore, let $x_1, x_2$ be the children of *x* in $S'$, and let $X = L(S'(x_1))$ and $Y = L(S'(x_2))$. Note that $\{X,Y\}$ is a partition of the children of *x* in *S*. Consider the trees $S^*|_X$ and $S^*|_Y$. We define another tree $\widehat{S}$ obtained by replacing the children of *x* in $S^*$ by $S^*|_X$ and $S^*|_Y$. More precisely, first observe that, by construction $L(S^*|_X)= X$ and $L(S^*|_Y)= Y$. Moreover, for any binary refinement $S^*$ of *S* it must hold that $L(S^*(x))$ is the set of children $\mathrm {ch}(x)$ in *S*. In particular, *x* is an ancestor in $S^*$ of every vertex in $S^*|_X$ as well as in $S^*|_Y$. Hence, we can safely replace the two subtrees $S^*(v_1)$ and $S^*(v_2)$ rooted at the two children $v_1,v_2$ of *x* in $S^*$ by $S^*|_X$ and $S^*|_Y$ (by defining the root of $S^*|_X$ and the root of $S^*|_Y$ as the two new children of *x*) to obtain another tree $\widehat{S}$ with $L(\widehat{S}) = L(S^*)$. By construction, $\widehat{S}$ is identical to $S^*$, except that the two subtrees below *x* are replaced by $S^*|_X$ and $S^*|_Y$. An example of the trees $S, S', S^*$ and $\widehat{S}$ is shown in Figure 4.

Let $\widehat{x}_1$ and $\widehat{x}_2$ be the children of *x* in $\widehat{S}$, with $L(\widehat{S}(\widehat{x}_1)) = X$ and $L(\widehat{S}(\widehat{x}_2)) = Y$. Clearly, $\widehat{S}(\widehat{x}_1)= S^*|_X$, resp., $\widehat{S}(\widehat{x}_2) =S^*|_Y$ is a binary refinement of $S'(x_1)$, resp., $S'(x_2)$. Moreover, $S^*|_{L(S^*)\setminus (X\cup Y)}$ is a binary refinement of $S'|_{L(S')\setminus (X\cup Y)}$. Taking the latter two arguments together, $\widehat{S}$ is a binary refinement of $S'$.

We proceed with showing that $\widehat{S}$ is a solution to $((T; t, \sigma ), S')$. To this end, we apply Prop. 1 and show that $\widehat{S}$ agrees with $\mathcal {R}(T; t,\sigma )$ and that $A({T},{\widehat{S}})$ is acyclic.

Let us first argue that $\widehat{S}$ agrees with $\mathcal {R}(T; t,\sigma )$. Observe first that since $\widehat{S}$ contains $S^*|_X$ and $S^*|_Y$ as subtrees, $\widehat{S}$ displays all triplets in $ab|c \in rt(S^*)$ with $a,b,c\in X$, or with $a,b,c\in Y$. Moreover, $\widehat{S}$ displays all triplets $ab|c \in rt(S^*)$ for which at least one of *a*, *b* and *c* is not contained in $X\cup Y$. The latter two arguments and ${\text {lca}}_{\widehat{S}}(X\cup Y)=x$ imply that $\widehat{S}$ displays all triplets $ab|c \in rt(S^*)$ except possibly those for which $lca_{\widehat{S}}(a,b,c) = x$. Let $R_x = \{ab|c \in rt(\widehat{S}) : lca_{\widehat{S}}(a,b,c) = x\}$. By the latter arguments, the only triplets in $rt(\widehat{S})$ that are not in $rt(S^*)$ are in $R_x$, i.e. $rt(\widehat{S}) \subseteq rt(S^*) \cup R_x$. By the definition of a good split refinement, $S'$ agrees with $\mathcal {R}(T; t,\sigma )$. Note that $R_x$ contains precisely those triplets *ab*|*c* for which either $a,b\in X$ and $c\in Y$ or $c\in X$ and $a,b\in Y$. This observation immediately implies that $R_x \subseteq rt(S')$. We thus have $rt(\widehat{S}) \subseteq rt(S^*) \cup rt(S')$ and since both $S^*$ and $S'$ agree with $\mathcal {R}(T; t,\sigma )$, it follows that $\widehat{S}$ agrees with $\mathcal {R}(T; t,\sigma )$.

We must now argue that $A({T},{\widehat{S}})$ is acyclic. Assume for contradiction that $A({T},{\widehat{S}})$ contains a cycle $C = (w_1, w_2, \ldots , w_k, w_1)$. Since $\widehat{S}$ is binary and agrees with $\mathcal {R}(T; t, \sigma )$, $\widehat{S}$ displays $\mathcal {R}(T; t, \sigma )$ and Theorem 1 implies that there is a reconciliation map from from $(T;t,\sigma )$ to $\widehat{S}$. By Lemma 3, $A({T},{\widehat{S}})$ does not contain self-loops and thus $k>1$ for $C = (w_1,\ldots , w_k, w_1)$. We will derive a contradiction by showing that $A({T},{S^*})$ contains a cycle. The proof is divided in a series of claims.

- Claim 1*If* $(u, v) \in E(A({T},{\widehat{S}}))$*and* $u, v \notin V(\widehat{S}(x))$, *then* $(u, v) \in E(A({T},{S^*}))$.
  Note that $\widehat{S}$ and $S^*$ are identical except for the subtree rooted at *x*. Thus, (*u*, *v*) is an A2-edge in $E(A({T},{\widehat{S}})$ if and only if it is an A2-edge in $A({T},{S^*})$ (assuming $u, v \notin V(\widehat{S}(x))$). Moreover, for all other edge types, notice that for every $w \in V(T)$ such that $\widehat{\mu }_{\widehat{S}}(w) \in V(\widehat{S}) \setminus V(\widehat{S}(x))$, we have $\widehat{\mu }_{\widehat{S}}(w) = \widehat{\mu }_{S^*}(w)$. Thus if (*u*, *v*) is an edge of $A({T},{\widehat{S}})$ with $u, v \notin V(\widehat{S}(x))$, then it is of one of the forms $(w, z), (\widehat{\mu }_{\widehat{S}}(w), z), (w, \widehat{\mu }_{\widehat{S}}(z))$ or $(\widehat{\mu }_{\widehat{S}}(w), \widehat{\mu }_{\widehat{S}}(z))$, where $(w, z) \in E(T)$. Since *T* does not change and by the previous argument, all four cases lead to $(u, v) \in E(A({T},{S^*}))$. This proves Claim 1.

To stress once again, since $\widehat{S}$ is binary and agrees with $\mathcal {R}(T; t,\sigma )$, it must display $\mathcal {R}(T; t,\sigma )$. Thus, we can apply Theorem 1 to conclude that there is a reconciliation map from $\widehat{S}$ to $(T; t,\sigma )$.

Now, let $Z = (V(T) \cup V(\widehat{S})) \setminus V(\widehat{S}(x))$. Observe that $Z = (V(T) \cup V(S^*)) \setminus V(S^*(x))$. If *C* does not contain a vertex of $V(\widehat{S}(x))$, then by Claim 1, every edge of *C* is also in $A({T},{S^*})$. Thus *C* is also a cycle in $A({T},{S^*})$, contradicting that it is acyclic. Therefore, we may assume that *C* contains at least one vertex from $V(\widehat{S}(x))$. On the other hand, assume that *C* does not contain a vertex of *Z*. Then all the vertices of *C* belong to $V(\widehat{S}(x))$. Since, as we argued before, $A({T},{\widehat{S}})$ does not contain self-loops, we conclude that every edge (*u*, *v*) of *C* is either an A1- or an A2-edge of $A({T},{\widehat{S}})$ that satisfies $v \prec _{\widehat{S}} u$. However, this implies that the edges of *C* cannot form a cycle; a contradiction. Therefore, *C* must contain vertices from both $V(\widehat{S}(x))$ and *Z*. Assume, without loss of generality, that $w_1 \in V(\widehat{S}(x))$ and $w_k \in Z$.

Now, *C* can be decomposed into a set of subpaths that alternate between vertices of $V(\widehat{S}(x))$ and of *Z*. More precisely, we say that a subpath $P = (w_i, w_{i+1}, \ldots , w_l)$ of *C*, where $1 \le i \le l \le k$, is a $V(\widehat{S}(x))$-subpath if $w_i, \ldots , w_l \in V(\widehat{S}(x))$. Similarly, we say that *P* is a *Z*-subpath if $w_i, \ldots , w_l \in Z$. Now, $C = (w_1, \ldots , w_k)$ is a concatenation of subpaths $P_1, P'_1, P_2, P'_2, \ldots , P_h, P'_h$ such that for $1 \le i \le h$, $P_i$ is a non-empty $V(\widehat{S}(x))$-subpath and $P'_i$ is a non-empty *Z*-subpath.

We want to show that $A({T},{S^*})$ contains a cycle. To this end, we will construct a cycle $C^*$ in $A({T},{S^*})$ such that $C^*$ is the concatenation of subpaths $P_1^*, P_1', \ldots , P_h^*, P_h'$, where each $P^*_i$ is a subpath of $A({T},{S^*})$ that replaces $P_i$. First notice that for each $1 \le i \le h$, all the edges of $P'_i$ are in $A({T},{S^*})$ by Claim 1. Therefore, every $P'_i$ is a path in $A({T},{S^*})$.

In what follows, we consider the $V(\widehat{S}(x))$-subpath $P_i = (w_p, w_{p+1}, \ldots , w_q)$, where $1 \le i \le h$ ($w_p = w_q$ may be possible if $P_i$ consists of a single vertex only). Notice that $w_{p - 1}$ and $w_{q+1}$ are in *Z* (where we define $w_{p-1} = w_k$ if $p = 1$ and $w_{q+1} = w_1$ if $p = k$). We construct a path $P^*_i = (w^*_1, \ldots , w^*_r)$ of $A({T},{S^*})$ such that $(w_{p-1}, w^*_1) \in E(A({T},{S^*}))$ and $(w^*_r, w_{q+1}) \in E(A({T},{S^*}))$.

To this end, we provide the following

- Claim 2 *The vertex x does not belong to* *C*.
  Let *Q* be a maximal topological sort of $A({T},{S})$ and let $Q'$ be a maximal topological sort of $A({T},{S'})$. By Lemma 6, $\mathcal {M}(Q) \subseteq \mathcal {M}(Q')$. Moreover, since $S'$ is a good split refinement, all the in-neighbors of *x* in $A({T},{S'})$ belong to *Q*. Since $\mathcal {M}(Q) \subseteq \mathcal {M}(Q')$, all the in-neighbors of *x* in $A({T},{S'})$ are also in $Q'$. This and maximality of $Q'$ implies that *x* is itself also in $Q'$. Let $\widehat{Q}$ be a maximal topological sort of $A({T},{\widehat{S}})$. Since $\widehat{S}$ can be obtained from a sequence of split refinements starting from $S'$, Lemma 6 implies that $\mathcal {M}(Q') \subseteq \mathcal {M}(\widehat{Q})$. In particular, $x \in \mathcal {M}(\widehat{Q})$. Lemma 4 implies that *x* cannot be contained in any cycle of $A({T},{\widehat{S}})$, which proves Claim 2.

Recalling (again) that $A({T},{\widehat{S}})$ does not contain self-loops, every edge (*u*, *v*) of $P_i$ is an A1- or A2-edge of $A({T},{\widehat{S}})$ and satisfies $v \prec _{\widehat{S}} u$. This implies that either $w_q \prec _{\widehat{S}} w_{q-1} \prec _{\widehat{S}} \ldots \prec _{\widehat{S}} w_p$, or that $w_p=w_q$. In either case, we have $w_q \preceq _{\widehat{S}} w_p$. By Claim 2, $w_p \ne x$. This and $w_p\in V(\widehat{S}(x))$ implies that $w_p \prec _{\widehat{S}} x$. By construction of $\widehat{S}$ we therefore have $L(\widehat{S}(w_p)) \subseteq X$ or $L(\widehat{S}(w_p)) \subseteq Y$. We will assume, without loss of generality, that $L(\widehat{S}(w_p)) \subseteq X$. Since $w_q \preceq _{\widehat{S}} w_p$, we have $L(\widehat{S}(w_q)) \subseteq L(\widehat{S}(w_p)) \subseteq X$. We now construct two important sets $X_p \subseteq X$ and $X_q \subseteq X$ that are quite helpful for our construction of a cycle $C^*$ in $A({T},{S^*})$.

- Claim 3*There exists a subset* $X_p \subseteq X$*such that* $w_p ={\text {lca}}_{\widehat{S}}(X_p)$*and* $(w_{p-1}, {\text {lca}}_{S^*}(X_p)) \in E(A({T},{S^*}))$.
  Since $w_p \in V(\widehat{S})$, the edge $(w_{p-1}, w_p)$ is either an A1-, A2- or A3-edge in $A({T},{\widehat{S}})$. Suppose first that $(w_{p-1}, w_p)$ is an A2-edge. Then $w_{p-1}$ is the parent of $w_p$ in $\widehat{S}$. Since $w_p \prec _{\widehat{S}} x$, this implies that $w_{p-1} \in V(\widehat{S}(x))$, contradicting $w_{p-1} \in Z$. Therefore, this case is not possible.
  Suppose that $(w_{p-1}, w_p)$ is an A1-edge defined by some $(u, v) \in E(T)$. Then $w_p\in V(\widehat{S})$ implies $w_p = \widehat{\mu }_{\widehat{S}}(v) = {\text {lca}}_{\widehat{S}}(\sigma _{T_{\mathcal {\overline{E}}}}(v))$ and we define $X_p = \sigma _{T_{\mathcal {\overline{E}}}}(v)$. We must prove that $(w_{p-1}, {\text {lca}}_{S^*}(X_p)) \in E(A({T},{S^*}))$. Since $(u, v) \in E(T)$ yields the A1-edge $(w_{p-1}, w_p)$ in $A({T},{\widehat{S}})$, we have $t(v)\in \{\odot , \mathfrak {s}\}$. Hence, (*u*, *v*) yields some A1-edge $(z, {\text {lca}}_{S^*}(X_p))$ in $A({T},{S^*})$ for some vertex *z*. In what follows, we show that $z=w_{p-1}$.
  If $w_{p-1} \in V(T)$, then $w_{p-1} = u$ and (*u*, *v*) defines the A1-edge $(u, \widehat{\mu }_{S^*}(v)) = (w_{p-1}, {\text {lca}}_{S^*}(X_p))$ in $A({T},{S^*})$. If $w_{p-1} \in V(\widehat{S})$, then $w_{p-1} = \widehat{\mu }_{S^*}(u)$. Since $w_{p-1} \in Z$, vertex $w_{p-1}$ must be a strict ancestor of *x* in $\widehat{S}$. This and the fact that $S^*$ and $\widehat{S}$ coincide except possibly in $S^*(x)$ and $\widehat{S}(x)$ implies that $\widehat{\mu }_{\widehat{S}}(u) = \widehat{\mu }_{S^*}(u) = w_{p-1}$. Hence, $(w_{p-1}, {\text {lca}}_{S^*}(X_p)) \in E(A({T},{S^*}))$.
  Finally, suppose that $(w_{p-1}, w_p)$ is an A3-edge defined by some $u \in V(T)$. Then $w_{p-1} = u$ and $w_p = \widehat{\mu }_{\widehat{S}}(u) = {\text {lca}}_{\widehat{S}}(\sigma _{T_{\mathcal {\overline{E}}}}(u))$, where $\sigma _{T_{\mathcal {\overline{E}}}}(u) \subseteq X$. Define $X_p = \sigma _{T_{\mathcal {\overline{E}}}}(u)$. Then $(w_{p-1}, w_p) = (u, {\text {lca}}_{\widehat{S}}(X_p))$ and $(u, \widehat{\mu }_{S^*}(u)) = (w_{p-1}, {\text {lca}}_{S^*}(X_p)) \in E(A({T},{S^*}))$. This proves Claim 3.

- Claim 4: *There exists a subset* $X_q \subseteq X$*such that* $w_{q} = {\text {lca}}_{\widehat{S}}(X_q)$*and* $({\text {lca}}_{S^*}(X_q), w_{q+1}) \in E(A({T},{S^*}))$.
  We show first that $w_{q+1} \in V(T)$. Assume, for contradiction, that $w_{q+1} \in V(\widehat{S})$. Since $(w_q,w_{q+1})$ is an edge of $A({T},{\widehat{S}})$ and since $w_{q} \in V(\widehat{S})$, the edge $(w_q,w_{q+1})$ is an A1- or A2-edge in $A({T},{\widehat{S}})$. However in both cases, because there are no self-loops, $w_{q+1} \prec _{\widehat{S}} w_q$ and thus, $w_{q+1}\in V(\widehat{S}(x))$ ; a contradiction to $w_{q+1} \in Z$. Hence, $w_{q+1} \in V(T)$. Therefore, $(w_q, w_{q+1})$ is either an A1- or A4-edge in $A({T},{\widehat{S}})$.
  Suppose first that $(w_q, w_{q+1})$ is an A1-edge of $A({T},{\widehat{S}})$ defined by some $(u, v) \in E(T)$. Then $(w_q, w_{q+1}) = (\widehat{\mu }_{\widehat{S}}(u), v)$, where $\widehat{\mu }_{\widehat{S}}(u) = {\text {lca}}_{\widehat{S}}(\sigma _{T_{\mathcal {\overline{E}}}}(u))$ and where $\sigma _{T_{\mathcal {\overline{E}}}}(u) \subseteq X$. Define $X_q = \sigma _{T_{\mathcal {\overline{E}}}}(u)$. Then $(w_q, w_{q+1}) = ({\text {lca}}_{\widehat{S}}(X_q), w_{q+1})$, and $(\widehat{\mu }_{S^*}(u), v) = ({\text {lca}}_{S^*}(X_q), w_{q+1})$ is an A1-edge of $A({T},{S^*})$.
  Suppose instead that $(w_q, w_{q+1})$ is an A4-edge of $A({T},{\widehat{S}})$ defined by some $(u, v) \in \mathcal {E}_T$ with $u=w_{q+1}$. Then $(w_q, w_{q+1}) = ({\text {lca}}_{\widehat{S}}(\widehat{\mu }_{\widehat{S}}(u), \widehat{\mu }_{\widehat{S}}(v)), u) = ({\text {lca}}_{\widehat{S}}(\sigma _{T_{\mathcal {\overline{E}}}}(u) \cup \sigma _{T_{\mathcal {\overline{E}}}}(v)), u)$. Define $X_q = \sigma _{T_{\mathcal {\overline{E}}}}(u) \cup \sigma _{T_{\mathcal {\overline{E}}}}(v)$. Hence, $w_q = {\text {lca}}_{\widehat{S}}(X_q)$, and since $w_q \in V(\widehat{S}(x))$, we must have $\sigma _{T_{\mathcal {\overline{E}}}}(u) \cup \sigma _{T_{\mathcal {\overline{E}}}}(v) \subseteq X$. Moreover, $({\text {lca}}_{S^*}(\widehat{\mu }_{S^*}(u), \widehat{\mu }_{S^*}(v)), u) = ({\text {lca}}_{S^*}(X_q), w_{q+1})$ is an A4-edge of $A({T},{S^*})$. This completes the proof of Claim 4.

- Claim 5: *Let* $X_p$*and* $X_q$*be subsets of* *X* *as defined in Claim 3 and 4. Then in* $A({T},{S^*})$, *there exists a path from* ${\text {lca}}_{S^*}(X_p)$ to ${\text {lca}}_{S^*}(X_q)$.
  By Claim 3 and 4 we have $w_p = {\text {lca}}_{\widehat{S}}(X_p)$ and ${\text {lca}}_{\widehat{S}}(X_q) = w_q$, respectively. As argued after the proof of Claim 2, we have ${\text {lca}}_{\widehat{S}}(X_q) = w_q \preceq _{\widehat{S}} w_p = {\text {lca}}_{\widehat{S}}(X_p)$. Because $\widehat{S}$ contains $S^*|_X$ as a rooted subtree, it follows that ${\text {lca}}_{S^*}(X_q) \preceq _{S^*} {\text {lca}}_{S^*}(X_p)$. Because of the A2-edges, there must be a path from ${\text {lca}}_{S^*}(X_p)$ to ${\text {lca}}_{S^*}(X_q)$ in $A({T},{S^*})$. This completes the proof of Claim 5.

We may now finish the argument. For each $1 \le i \le h$, we let $P^*_i$ be the path obtained from Claim 5. We claim that by concatenating the paths $P^*_1, P'_1, P^*_2, P'_2, \ldots , P^*_h, P'_h$ in $A({T},{S^*})$, we obtain a cycle. We have already argued that each $P^*_i$ and each $P'_i$ is a path in $A({T},{S^*})$. The rest follows from Claim 4, since it implies that for each $1 \le i \le h$, the last vertex of $P^*_i$ has the first vertex of $P'_i$ as an out-neighbor, and the last vertex of $P'_i$ has the first vertex of $P^*_{i+1}$ as an out-neighbor (where $P^*_{h+1}$ is defined to be $P^*_1$). We have thus found a cycle in $A({T},{S^*})$, a contradiction to the acyclicity of $A({T},{S^*})$.

Hence, $A({T},{\widehat{S}})$ is acyclic. This and the fact that $\widehat{S}$ displays $\mathcal {R}(T;t,\sigma )$ implies that $\widehat{S}$ is a solution to $((T; t, \sigma ), S')$. Therefore, $((T; t, \sigma ), S')$ admits a solution. $\square$

### A.5 Proof of Theorem 5

Before proving Theorem 5, we provide a result that shows how maximal topological sorts and strict ancestors of vertices in *S* are related w.r.t. split refinements of *S*.

#### Lemma 7

*Let* *Q* *be a maximal topological sort of* $A({T},{S})$. *If there exists a good split refinement* $S'$*of* *S* *at a cherry* *x*, *then every strict ancestor of* *x* *in* *S* *and* $S'$*is in* $\mathcal {M}(Q)$.

#### Proof

Let $S'$ be a good split refinement of *S* at *x*. By construction, the sets of ancestors of *x* in *S* and $S'$ are equal.

Assume that there is a strict ancestor *y* of *x* that is not in *Q*. Due to the A2-edges in $A({T},{S})$ there is a directed path *P* from *y* to *x* in $A({T},{S})$. Lemma 4 implies that none of the vertices along this path *P* are contained in $\mathcal {M}(Q)$. Since *y* is a strict ancestor of *x* in *S*, we can conclude that the parent *p*(*x*) of *x* in *S* is not contained in $\mathcal {M}(Q)$. Again, due to the A2-edges of $A({T},{S'})$, the pair (*p*(*x*), *x*) is an edge in $A({T},{S'})$ and hence, *p*(*x*) is an in-neighbor of *x* in $A({T},{S'})$. However, since $S'$ is a good split refinement of *S*, all the in-neighbors of *x* in $A({T},{S'})$ must, by definition, belong to $\mathcal {M}(Q)$; a contradiction. Thus, every strict ancestor *y* of *x* in *S* and $S'$ is in $\mathcal {M}(Q)$. $\square$

In what follows, when we ask whether a fixed *x* admits a good split refinement, we can first check whether all of its ancestors are in *Q*, where *Q* is a maximal topological sort of $A({T},{S})$. If this is not the case, then, by contraposition of Lemma 7, we may immediately conclude that there is no good split refinement at *x*.

Otherwise, we investigate *x* further.

We are now in a position to prove Theorem 5.

#### Proof of Theorem 5

In what follows, put $G {:}{=}G((T; t, \sigma ), S, x)$. Suppose that there exists a good split refinement $S'$ of *S*. Let *x* be the cherry of *S* that was refined from *S* to $S'$, and let $x_1, x_2$ be the children of *x* in $S'$. Let *Q* be a maximal topological sort of $A({T},{S})$. By Lemma 7, every strict ancestor of *x* in *S* is in *Q*. Let $A = L(S(x_1))$ and $B = L(S(x_2))$. We claim that for any pair $a \in A, b \in B$, $ab \notin E(G)$.

Assume for contradiction that there is an edge *ab* with $a \in A, b \in B$. We treat each possible edge type separately.

(C1): Suppose that $ab \in E(G)$ because there exists $c \in L(S(x))$ such that $ab|c \in \mathcal {R}(T; t,\sigma )$. Because $a \in A$ and $b \in B$ and by construction of $S'$, we either have $ac|b \in rt(S')$ if $c \in A$, or $bc|a \in rt(S')$ if $c \in B$. In either case, $S'$ does not agree with $\mathcal {R}(T; t,\sigma )$, contradicting that $S'$ is a good split refinement.

**(C2)**: Suppose instead that $ab \in E(G)$ because there exists an edge $(u, v) \in E(T)$ with $t(u) \in \{\mathfrak {d}, \mathfrak {t}\}$, $u \notin \mathcal {M}(Q)$, $t(v) = \mathfrak {s}$ and $a,b \in \sigma _{T_{\mathcal {\overline{E}}}}(v)$. By construction of $S'$ and due to the choice of $A = L(S(x_1))$ and $B = L(S(x_2))$, we have $\widehat{\mu }_{S'}(v) = lca_{S'}(\sigma _{T_{\mathcal {\overline{E}}}}(v)) \succeq _{S'} lca_{S'}(a,b) = x$. If $\widehat{\mu }_{S'}(v) = x$, then $(u, \widehat{\mu }_{S'}(v)) = (u, x)$ is an A1-edge of $A({T},{S'})$. Thus, *x* has in-neighbor *u* in $A({T},{S'})$ such that $u \notin \mathcal {M}(Q)$, which contradicts that $S'$ is a good split refinement. So assume that $\widehat{\mu }_{S'}(v) \succ _{S'} x$. In this case, $(u, \widehat{\mu }_{S'}(v))$ is an A1-edge of $S'$, and by Lemma 5, $(u, \widehat{\mu }_{S'}(v)) \in E(A({T},{S}))$. Since $u \notin \mathcal {M}(Q)$, we must have $\widehat{\mu }_{S'}(v) \notin \mathcal {M}(Q)$. Since $\widehat{\mu }_{S'}(v) \succ _S x$, we obtain a contradiction to Lemma 7.

**(C3)**: Suppose that $ab \in E(G)$ because there exists an edge $(u, v) \in E(T)$ with $t(u) = t(v) = \mathfrak {s}$, $\widehat{\mu }_{S}(u) = x$ and $a,b \in \sigma _{T_{\mathcal {\overline{E}}}}(v)$. Note, since ${\text {lca}}_S(a,b)=x$ and $a,b \in \sigma _{T_{\mathcal {\overline{E}}}}(v)$, it must hold that $x\preceq _S\widehat{\mu }_S(v)$. Moreover, $t(u) = t(v) = \mathfrak {s}$ implies that *u* and *v* are contained in the same connected component of $T_{\mathcal {\overline{E}}}$. This and $v\prec _{T_{\mathcal {\overline{E}}}} u$ implies $\sigma _{T_{\mathcal {\overline{E}}}}(v)\subseteq \sigma _{T_{\mathcal {\overline{E}}}}(u)$. Hence, $\widehat{\mu }_S(v) \preceq _S \widehat{\mu }_S(u)$. Now, $x\preceq _S\widehat{\mu }_S(v) \preceq _S \widehat{\mu }_S(u) = x$ implies $\widehat{\mu }_S(v) =\widehat{\mu }_S(u) = x$. Therefore, $(\widehat{\mu }_S(u), \widehat{\mu }_S(v)) = (x, x)$ is an A1-edge of $A({T},{S})$, and it follows that $x \notin \mathcal {M}(Q)$ (a vertex with a self-loop cannot never be added to a maximal topological sort). Moreover, because $a, b \in \sigma _{T_{\mathcal {\overline{E}}}}(v)$ and $a \in A = L(S(x_1))$ and $b\in B = L(S(x_2))$, it holds that $\widehat{\mu }_{S'}(u) = \widehat{\mu }_{S'}(v) = x$. Hence (*x*, *x*) is an A1-edge of $A({T},{S'})$ as well, and *x* has an in-neighbor not in *Q* (namely *x* itself). This contradicts the assumption that $S'$ is a good split refinement.

**(C4)**: Suppose that $ab \in E(G)$ because there is a vertex $u \in V(T) \setminus \mathcal {M}(Q)$ such that $t(u) \in \{\mathfrak {d}, \mathfrak {t}\}$ and $a,b \in \sigma _{T_{\mathcal {\overline{E}}}}(u)$. The reasoning is similar to Case (C2). That is, we must have $p {:}{=}\widehat{\mu }_{S'}(u) = lca_{S'}(\sigma _{T_{\mathcal {\overline{E}}}}(u)) \succeq _{S'} lca_{S'}(a, b) = x$. Now, $A({T},{S'})$ contains the A3-edge (*u*, *p*). We cannot have $p = x$ because $u \notin \mathcal {M}(Q)$ and $S'$ is a good split refinement of *S*. Thus $p \succ _{S'} x$. In this case, $(u, p) \in E(A({T},{S}))$ by Lemma 5. Thus *p* cannot be in $\mathcal {M}(Q)$, which contradicts Lemma 7.

We have thus shown that *ab* cannot exist for any pair $a\in A$ and $b\in B$. Since *A* and *B* form a partition of *V*(*G*), the graph *G* must be disconnected.

Conversely, suppose that there exists a cherry *x* of *S* such that *G* is disconnected and such that every strict ancestor of *x* in *S* is in *Q*. Let (*A*, *B*) be any disconnected bipartition of *G*. Furthermore, let $S'$ be the split refinement of *S* obtained by splitting the children of *x* into *A* and *B* and let $x_1, x_2$ be the two children of *x* in $S'$. W.l.o.g. assume that $x_1$ and $x_2$ is the ancestor of the leaves in *A* and *B*, respectively. We claim that $S'$ is a good split refinement.

Let us first argue that $S'$ agrees with $\mathcal {R}(T; t, \sigma )$. Assume for contradiction that $S'$ displays a triplet *ac*|*b*, but that $ab|c \in \mathcal {R}(T; t, \sigma )$. By assumption, *S* agrees with $\mathcal {R}(T; t, \sigma )$, so $ac|b \in rt(S') \setminus rt(S)$. This implies that ${\text {lca}}_{S'}(a, b) = {\text {lca}}_{S'}(c, b) = x$. W.l.o.g we may assume that $a, c \in A$ and $b \in B$. However, Condition (C1) implies that we have the edge $ab \in E(G)$, contradicting that (*A*, *B*) forms a disconnected bipartition. Therefore, $S'$ agrees with $\mathcal {R}(T; t, \sigma )$.

It remains to show that all in-neighbors of *x* in $A({T},{S'})$ are contained in $\mathcal {M}(Q)$. Assume, for contradiction, that there is an edge $(p, x) \in E(A({T},{S'}))$ such that $p \notin \mathcal {M}(Q)$. Since $x\in V(S')$, the edge (*p*, *x*) it either an A1-, A2- or A3-edge in $A({T},{S'})$. As it is now our routine, we check several cases separately.

- *Case:* (*p*, *x*) *is an A1-edge and* $p \ne x$.
  In this case (*p*, *x*) is defined by some edge $(u, v) \in E(T)$. Suppose that $(p, x) = (\widehat{\mu }_{S'}(u), \widehat{\mu }_{S'}(v))$. Since $p \ne x$, *p* is a strict ancestor of *x* in $S'$, and hence also in *S*. This is not possible, since we assume that every strict ancestor of *x* in *S* belongs to *Q* (whereas here we suppose $p \notin \mathcal {M}(Q)$). We deduce that $(p, x) = (u, \widehat{\mu }_{S'}(v))$. Therefore, $u \notin \mathcal {M}(Q)$, $t(u) \in \{\mathfrak {d}, \mathfrak {t}\}$ and $t(v) = \mathfrak {s}$. Moreover, since $\widehat{\mu }_{S'}(v) = x$ and *x* has only the two children $x_1$ and $x_2$ in $S'$, we can conclude there are $a,b \in \sigma _{T_{\mathcal {\overline{E}}}}(v)$ such that $a \preceq _{S'} x_1$ and $b \preceq _{S'} x_2$, i.e. $a \in A, b \in B$. The latter two arguments imply that Condition (C2) is satisfied for *a* and *b* and, therefore, $ab\in E(G)$; a contradiction to (*A*, *B*) are forming a disconnected bipartition.
- *Case:* (*p*, *x*) *is an A1-edge and* $p = x$.
  In this case, $(p, x) = (x, x) = (\widehat{\mu }_{S'}(u), \widehat{\mu }_{S'}(v))$ is defined by some edge (*u*, *v*) of *T*. Since *x* is an internal vertex of $S'$, we must have $t(u) = t(v) = \mathfrak {s}$. Since $L(S(x)) = L(S'(x))$ and *x* is a cherry in *S*, we also have $(\widehat{\mu }_S(u), \widehat{\mu }_S(v)) = (x, x)$. Moreover because $\widehat{\mu }_{S'}(v) = x = {\text {lca}}_{S'}(x_1,x_2)$, there must exist distinct *a*, *b* with $a\prec _{S'}x_1$ and $b\prec _{S'}x_2$ such that $a,b \in \sigma _{T_{\mathcal {\overline{E}}}}(v)$. Thus, $a \in A, b \in B$. Moreover *ab* satisfies the Condition (C3). Thus, $ab\in E(G)$; a contradiction to our assumption that (*A*, *B*) forms a disconnected bipartition.
- *Case:* (*p*, *x*) *is an A2-edge.*
  This case is not possible, since the parent of *x* is the same in *S* and $S'$, and we assume that all strict ancestors of *x* in *S* are in *Q*.
- *Case:* (*p*, *x*) *is an A3-edge.*
  In this case, $(p, x) = (u, \widehat{\mu }_{S'}(u))$ is defined by a vertex $u \in V(T)$ such that $t(u) \in \{\mathfrak {d}, \mathfrak {t}\}$ and $\widehat{\mu }_{S'}(u) = x$. Since $u = p$ and, by assumption $p \notin \mathcal {M}(Q)$, we have $u \in V(T) \setminus \mathcal {M}(Q)$.
  As in the A1-case, there must be $a, b \in \sigma _{T_{\mathcal {\overline{E}}}}(u)$ such that $a \in A, b \in B$. Then *ab* should be an edge of *G* because of Condition (C4), a contradiction.

We have shown that the (*p*, *x*) edge cannot exist. Therefore in $A({T},{S'})$, all the in-neighbors of *x* are in *Q*. Since $S'$ also agrees with *R*, it follows that splitting the children of *x* into (*A*, *B*) forms a good split refinement at *x*. $\square$

### A.6 Proof of Lemma 2

#### Proof of Lemma 2

To compute $\mathcal {R}(T; t, \sigma )$ as in Def. 1 we can proceed as follows: We first compute the ${\text {lca}}_{T_{\mathcal {\overline{E}}}}$’s for every pair of vertices within the connected components of $T_{\mathcal {\overline{E}}}.$ This task can be done in constant time for each pair of vertices after linear preprocessing of the trees in $T_{\mathcal {\overline{E}}}$ [53, 54]. Thus, we end in an overall time complexity of $O(n^2)$ to compute all ${\text {lca}}_{T_{\mathcal {\overline{E}}}}$’s between the leaves of *T*. We now compute the distance from the root $\rho _{{{\widetilde{T}}}}$ to all other vertices in $V({{\widetilde{T}}})$ for every connected component ${{\widetilde{T}}}$ of $T_{\mathcal {\overline{E}}}$. The latter can be done for each individual connected component ${{\widetilde{T}}}$ via Dijkstra’s algorithm in $O(|V({{\widetilde{T}}})|^2)$ time. As this must be done for all connected components of $T_{\mathcal {\overline{E}}}$ and since $\sum _{{{\widetilde{T}}}} |V({{\widetilde{T}}})|^2 \le (\sum _{{{\widetilde{T}}}} |V({{\widetilde{T}}})|)^2 = |V(T)|^2$ we end in time $O(|V(T)|^2) = O(n^2)$ to compute the individual distances. Now, for all three distinct leaves *a*, *b*, *c* within the connected components of $T_{\mathcal {\overline{E}}}$, we compare the relative order of $x={\text {lca}}_{T_{\mathcal {\overline{E}}}}(a,b)$, $y={\text {lca}}_{T_{\mathcal {\overline{E}}}}(a,c)$, and $z={\text {lca}}_{T_{\mathcal {\overline{E}}}}(b,c)$ which can be done directly by comparing the distances $d_{{{\widetilde{T}}}}(\rho _{{{\widetilde{T}}}},x)$, $d_{{{\widetilde{T}}}}(\rho _{{{\widetilde{T}}}},y)$ and $d_{{{\widetilde{T}}}}(\rho _{{{\widetilde{T}}}},z)$. It is easy to see that at least two of the latter three distances must be equal. Hence, as soon as we have found that two distances are equal but distinct from the third, say $d_T(\rho _T,x)\ne d_T(\rho _T,y)=d_T(\rho _T,z)$, we found the triplet *ab*|*c* that is displayed by ${{\widetilde{T}}}$. If, in addition, $t(z)=\mathfrak {s}$ and $\sigma (a), \sigma (b), \sigma (c)$ are pairwise distinct, then we add $\sigma (a)\sigma (b)|\sigma (c)$ to $\mathcal {R}(T; t, \sigma )$. The latter tasks can be done in constant for every triplet *a*, *b*, *c*. Since there are at most $$\left( {\begin{array}{c}n\\ 3\end{array}}\right) =O(n^3)$$ triplets in *T*, we end in an overall time-complexity $O(n^3)$ to compute all triplets displayed by *T* that satisfy Def. 1(1).

Now we proceed to construct for all transfer edges $(u,v)\in \mathcal {E}_T$ the triplets $\sigma (a)\sigma (b)|\sigma (c)$ for all $a,b\in L(T_{\mathcal {\overline{E}}}(u))$ and $c\in L(T_{\mathcal {\overline{E}}}(v))$ as well as for all $c\in L(T_{\mathcal {\overline{E}}}(u))$ and $a,b\in L(T_{\mathcal {\overline{E}}}(v))$ with $\sigma (a), \sigma (b), \sigma (c)$ being pairwise distinct. To this end, we need to compute $L(T_{\mathcal {\overline{E}}}(w))$ for all $w\in V(T)$. We may traverse every connected component ${{\widetilde{T}}}$ of $T_{\mathcal {\overline{E}}}$ from the root $\rho _{T_{\mathcal {\overline{E}}}}$ to each individual leaf and and for each vertex *w* along the path from $\rho _{T_{\mathcal {\overline{E}}}}$ to a leaf *l*, we add the leaf *l* to $L(T_{\mathcal {\overline{E}}}(w))$. As there are precisely $|L({{\widetilde{T}}})|$ such paths, each having at most $|V({{\widetilde{T}}})|\in O(|L({{\widetilde{T}}})|)$ vertices, we end in $O(|L({{\widetilde{T}}})|^2)$ time to compute $L(T_{\mathcal {\overline{E}}}(w))$ for all $w\in V({{\widetilde{T}}})$. As this step must be repeated for all connected components ${{\widetilde{T}}}$ of $T_{\mathcal {\overline{E}}}$ we end, by the analogous arguments as in the latter paragraph, in $\sum _{{{\widetilde{T}}}} O(|L({{\widetilde{T}}})|^2) = O(n^2)$ time to compute $L(T_{\mathcal {\overline{E}}}(w))$ for all $w\in V(T)$. Now, for every transfer edge $(u,v)\in \mathcal {E}_T$ the triplets $\sigma (a)\sigma (b)|\sigma (c)$ (with $\sigma (a), \sigma (b), \sigma (c)$ being pairwise distinct) are added to $\mathcal {R}(T; t, \sigma )$ for all $a,b\in L(T_{\mathcal {\overline{E}}}(u))$ and $c\in L(T_{\mathcal {\overline{E}}}(v))$ as well as for all $c\in L(T_{\mathcal {\overline{E}}}(u))$ and $a,b\in L(T_{\mathcal {\overline{E}}}(v))$. Note, none of the trees $T_{\mathcal {\overline{E}}}$ contains transfer edges. Moreover, for each transfer edge (*u*, *v*) we have, by Axiom (O3), $\sigma _{T_{\mathcal {\overline{E}}}}(v)\cap \sigma _{T_{\mathcal {\overline{E}}}}(u) = \emptyset$. The latter two arguments imply that, for each transfer edge (*u*, *v*), precisely $$\left( {\begin{array}{c}|\sigma (L(T_{\mathcal {\overline{E}}}(v)))|\\ 2\end{array}}\right) |\sigma (L(T_{\mathcal {\overline{E}}}(u)))|+ \left( {\begin{array}{c}|\sigma (L(T_{\mathcal {\overline{E}}}(u)))|\\ 2\end{array}}\right) |\sigma (L(T_{\mathcal {\overline{E}}}(v)))|$$ triplets are added.

Now, let $\mathcal {T} = \{T_1, T_2, \ldots , T_k\}$ be the set of trees in the forest $T_{\mathcal {\overline{E}}}$. For each $i \in \{1, \ldots , k\}$, define $n_i = |L(T_i)|$. Let us write $T_i \rightarrow T_j$ if there exists a transfer edge $(u, v) \in \mathcal {E}_T$ satisfying $u \in V(T_i), v \in V(T_j)$. It is easy to verify that there is exactly one transfer edge connecting two distinct connected components of $T_{\mathcal {\overline{E}}}$, as otherwise, some root vertex of some $T_j$ would have in-degree 2 or more in *T* (since all transfer edges go from a vertex to a root of another subtree of $T_{\mathcal {\overline{E}}}$). For each transfer edge $(u, v) \in \mathcal {E}_T$, where $u \in V(T_i)$ and $v \in V(T_j)$, we can bound the number of added triplets by $$\left( {\begin{array}{c}|\sigma _{T_{\mathcal {\overline{E}}}}(u)|\\ 2\end{array}}\right) |\sigma _{T_{\mathcal {\overline{E}}}}(v)|+ \left( {\begin{array}{c}|\sigma _{T_{\mathcal {\overline{E}}}}(v)|\\ 2\end{array}}\right) |\sigma _{T_{\mathcal {\overline{E}}}}(u)| \le n_i^2 n_j + n_i n_j^2$$. The total number of triplets considered is then at most

$$\begin{aligned}&\sum _{T_i \in \mathcal {T}} \sum _{T_j : T_i \rightarrow T_j} \left( n_i^2n_j + n_j^2n_i \right) \\&\quad = \sum _{T_i \in \mathcal {T}} n_i^2 \sum _{T_j : T_i \rightarrow T_j} n_j + \sum _{T_i \in \mathcal {T}} n_i \sum _{T_j : T_i \rightarrow T_j} n_j^2 \\&\quad \le \sum _{T_i \in \mathcal {T}} n_i^2 \cdot n + \sum _{T_i \in \mathcal {T}} n_i \cdot n^2 \\&\quad = n \sum _{T_i \in \mathcal {T}} n_i^2 + n^2 \sum _{T_i \in \mathcal {T}} n_i \le 2n^3 \in O(n^3) . \end{aligned}$$

In the latter approximation, we have used the fact that distinct trees $T_i$ and $T_j$ have disjoint sets of leaf sets (cf. [4, Lemma 1]). Thus, $\sum _{T_i \in \mathcal {T}} n_i \le n$ and $\sum _{T_j : T_i \rightarrow T_j} n_i \le n$. In summary, $\mathcal {R}(T; t, \sigma )$ can be computed in $O(n^3)$ time.

Finally, note that if $(T; t, \sigma )$ is binary such that all inner vertices are labeled as speciation $\mathfrak {s}$ and for all two distinct leaves $x,y\in L(T)$ we have $\sigma (x)\ne \sigma (y)$, then $$|\mathcal {R}(T; t, \sigma )|=\left( {\begin{array}{c}n\\ 3\end{array}}\right) \in O(n^3)$$. Hence, the boundary $O(n^3)$ can indeed be achieved and thus, the worst-case runtime is $\Theta (n^3)$. $\square$

### A.7 Proof of Theorem 6

#### Proof of Theorem 6

We first prove the correctness of the algorithm. Algorithm 2 takes as input a labeled gene tree $(T;t,\sigma )$. First $\mathcal {R}(T;t,\sigma )$ is computed and the star tree *S* (which clearly agrees with $\mathcal {R}(T;t,\sigma )$) will be furthermore refined. Moreover, *S* contains at this point of computation only one cherry, namely the root *r* of *S*, and $G((T;t,\sigma ),S,r)$ is computed. Now, in each step of the *while*-loop it is first checked if *S* is non-binary and if in one of the previous steps a good split refinement has been found. In this case, it is first checked (*for*-loop) if there are non-binary cherries of the current tree *S* for which all strict ancestors are contained in $\mathcal {M}(Q)$ with *Q* being the maximal topological sort of $A({T},{S})$. If this is not the case for all non-binary cherries, the *while*-loop terminates according to Lemma 7 and the algorithm correctly outputs *“No time-consistent species tree exists”*. Contrary, if there is a non-binary cherry *x* for which all strict ancestors are contained in $\mathcal {M}(Q)$, then it is checked whether we have not found already a good split for *S* and if $G((T;t,\sigma ),S,x)$ is disconnected. In this case, we can apply Theorem 5 to conclude that there is a good split refinement for *S* at *x* which is computed in the subsequent step. If, however, for all non-binary cherries $G((T;t,\sigma ),S,x)$ is connected, the algorithm correctly outputs *“No time-consistent species tree exists”* according to Theorem 5. Finally, if in each step of the *while*-loop we have found a good split refinement and *S* does not contain a non-binary cherry, then *S* must, by construction, be binary. In this case, repeated application of Theorem 3 shows that the final binary tree *S* is a solution to the underlying GTC instance and the algorithm correctly returns *S*. Thus, Algorithm 2 correctly computes a time-consistent binary species tree for $(T;t,\sigma )$, if one exists.

We next analyze the running time of the algorithm. Let $\Sigma = \sigma (L(T))$ be the set of species. We will frequently use the fact that $|\Sigma | \le n$. The main challenge in optimizing this algorithm is to be able to efficiently construct and update the $G((T;t,\sigma ), S, x)$ graphs. We will save this analysis for the end of the proof, and will ignore the time spent on graph updates for now.

We will assume that $\sigma _{T_{\mathcal {\overline{E}}}}(u)$ is computed and stored for each $u \in V(T)$. As argued in the proof of Lemma 2, this can be done in time $O(n^2)$. Also by Lemma 2, the triplet set $\mathcal {R}(T; t, \sigma )$ can be computed in $O(n^3)$ time. Since every iteration of the main *while* loop adds a new binary vertex in *S*, the loop will be executed *O*(*n*) times (since a binary tree on $|\Sigma | \le n$ leaves has *O*(*n*) internal vertices). By [4, Lemma 3], computing $\widehat{\mu }_{T,S}$ can be done in time O($n\log (|\Sigma |))=O(n\log (n))$. By [4, Thm. 6], the auxiliary graph $A({T},{S})$ can be computed in $O(|V(T)|\log (|V(S)|))$ time. Since $O(|V(T)|) = O(n)$ and $O(|V(S)|) = O(n)$, the latter task can be done in $O(n\log (n))$ time. Construction of *Q* can be done in time $O(|V(A({T},{S}))| + |E(A({T},{S}))|) = O(n)$ using the techniques of [52] and by observing that the edges in $E(A({T},{S}))$ cannot exceed $|E(T)|+|E(S)|=O(n)$.

In each pass of the main *while* loop, we iterate through *O*(*n*) non-binary cherries. Let $c_1, \ldots , c_k$ be the non-binary cherries of *S*, assuming that each auxiliary $G((T;t,\sigma ), S, c_i)$ graph is already pre-computed. Since $c_1, \ldots , c_k$ are cherries of *S*, the sets in $\{L(S(c_1)), L(S(c_2)), \ldots L(S(c_k))\}$ must be pairwise disjoint. Denoting $n_i = |L(S(c_i))|$, $1 \le i \le k$, we thus observe that $\sum _{i = 1}^{k}n_i \le n$. In the worst case, we go through every cherry and check connectedness in time $O(n_i^2)$ on each graph $G((T;t,\sigma ), S, c_i)$ via “classical” breadth-first search. Thus in one iteration of the main *while* loop, the total time spent on connectedness verification is $O(\sum _{i = 1}^{k}n_i^2) = O(n^2)$. When we apply a split refinement, we compute $\widehat{\mu }_{T,S}(u)$ for all $u\in V(T)$, $A({T},{S})$ and *Q* at most once per *while* iteration, each operation being feasible in time $O(n \log n)$. To be more precise, as soon as we have found a good split refinement we put Has_GoodSplit=TRUE. Hence, the *if*-condition (Line 11) will then not be satisfied, and we will not recompute the values $\widehat{\mu }_{T,S}(u)$, $A({T},{S})$ and *Q* again for the remaining non-binary cherries *x* of *S* within the *for*-loop (Line 10). As there are *O*(*n*) iterations, the time spent on operations other than graph construction and updates is $O(n^3)$.

Let us now argue that the total time spent on the auxiliary $G((T;t,\sigma ), S, x)$ graph updates can be implemented to take time $O(n^3)$. To this end, we maintain a special data structure that, for each 2-element subset $\{a,b\} \subseteq \Sigma$, remembers the members of $\Sigma \cup V(T)$ that may cause *ab* to be an edge in the auxiliary graphs. We describe this in more detail. For a certain species tree *S*, we say that $a,b \in L(S)$ are *siblings* if *a* and *b* have the same parent in *S*. For any two siblings *a*, *b* of *S*, define

$$\begin{aligned} l_1(a,b)&= \{c \in \Sigma :c \text{ is } \text{ a } \text{ sibling } \text{ of } a \text{ and } b, \text{ and } \\&\quad ab|c \in \mathcal {R}(T;t,\sigma )\} \\ l_2(a,b)&= \{u \in V(T) :\exists v \in V(T) \hbox { such that}\ (u,v) \\&\quad \text{ satisfies } \text{(C2) }\} \\ l_3(a,b)&= \{u \in V(T) :\exists v \in V(T) \hbox { such that}\ (u,v) \\&\quad \text{ satisfies } \text{(C3) } \text{ and } ab\in \mathrm {ch}(x)\}\\ l_4(a,b)&= \{u \in V(T) :u \text{ satisfies } \text{(C4) } \} \end{aligned}$$

Note that if we have access to each of the four $l_i$ sets for any siblings *a*, *b*, then we may construct a graph $G {:}{=}G((T;t,\sigma ), S, x)$ in time $O(n^2)$. Indeed, to decide whether there is an edge *ab* in $G((T;t,\sigma ), S, x)$, it suffices to check whether one $l_i(a,b)$, $1\le i \le 4$, is non-empty, a task that can done in constant time for each two vertices $a,b\in \Sigma$. Since there are $O(n^2)$ pairs to check, the graph construction takes time $O(n^2)$. After each binary refinement, there are two new graphs to construct (one for each child of the new binary vertex), and since there are $O(n^2)$ iterations, the total time spent for constructing graphs will be $O(n^3)$. The main challenge is to update the $l_i$ sets after a binary refinement, which we now analyze.

We thus show how to maintain these four sets for each pair of siblings as *S* undergoes split refinements, starting with the initial star tree *S*. With *S* being a star tree, the set $l_1(a,b)$ can be constructed in time $O(n^3)$ for all *a*, *b* by iterating through $\mathcal {R}(T;t,\sigma )$ once, and each $l_i(a,b)$, $i \in \{2,3,4\}$ can be constructed in time $O(n^3)$ by first constructing $A({T},{S})$ with its maximal topological sort *Q* and, for each *a*, *b* pair, checking every vertex and edge of *T* for conditions (C2), (C3) and (C4). It is easy to see that each condition can be checked in constant time per edge or vertex.

Now assume inductively that $l_1,l_2,l_3$ and $l_4$ are known for each pair of siblings of *S*. Assume that we perform a split refinement at some cherry *x* of *S*, yielding a tree $S'$ in which $x_1, x_2$ are the new children of *x*. We then need to compute $G((T;t,\sigma ), S, x_1)$ and $G((T;t,\sigma ), S, x_2)$. Denote $X_1 = L(S'(x_1))$ and $X_2 = L(S'(x_2))$, let *Q* (resp. $Q'$) be a maximal topological sort of $A({T},{S})$ (resp. $A({T},{S'})$). We describe how each $l_i$ update is done, and argue on the total time spent on each update type (by total time, we mean summing the time over every iteration of the *for* loop).

- (The $l_1$ set). For $a,b \in X_1$, we may need to remove *c* from $l_1(a,b)$ if $c \in X_2$ since it is not a sibling of *a* and *b* anymore. Thus, for each $a,b \in X_1$ and each $c \in X_2$, we remove *c* from $l_1(a,b)$ if present (and we do the same for each $a'b' \in X_2, c' \in X_1$). Therefore, each time that a pair $a,b \in \Sigma$ gets separated from some $c \in \Sigma$ during the species tree construction, we need *O*(1) time to remove *c* from $l_1(a, b)$. Importantly, this separation occurs at most once for each triplet $\{a,b,c\}$ during the whole algorithm execution. Therefore, in total we spend time *O*(1) on $l_1$ for each distinct $a,b,c \in \Sigma$, and so the total time spent on updating $l_1$ is $O(n^3)$.
- (The $l_2$ set). Let $a,b \in X_i$, $i \in \{1,2\}$. Recall that we must have $u \in l_2(a,b)$ if there is $(u, v) \in E(T)$ such that $t(u) \in \{\mathfrak {d}, \mathfrak {t}\}, u \notin \mathcal {M}(Q')$, $t(v) = \mathfrak {s}$ and $\{a, b\} \subseteq \sigma _{T_{\mathcal {\overline{E}}}}(v)$. Since $\mathcal {M}(Q) \subseteq \mathcal {M}(Q')$ and *t* does not change when refining, after one binary refinement we can only remove elements from $l_2(a, b)$, and never insert new elements. That is, we only need to remove some *u* from $l_2(a, b)$ if $u \notin \mathcal {M}(Q)$ but $u \in \mathcal {M}(Q')$. Therefore, when computing $Q'$ and adding a new element $u \in \mathcal {M}(Q') \setminus \mathcal {M}(Q)$, we remove *u* from each $l_2(a, b)$ that contains *u*. This takes time $O(n^2)$ every time we add a new element in the maximal topological sort, and this is enough to keep $l_2$ up-to-date. Importantly, each vertex of *T* gets added at most once in *Q* during the execution of the whole algorithm. Therefore, maintaining $l_2$ consumes total time $O(n^3)$ in total.
- (The $l_4$ set). Let $a,b \in X_i$, $i \in \{1,2\}$. Recall that we must have $u \in l_4(a,b)$ if $u \in V(T)$, $t(u) \in \{\mathfrak {d}, \mathfrak {t}\}, u \notin \mathcal {M}(Q')$, and $\{a, b\} \subseteq \sigma _{T_{\mathcal {\overline{E}}}}(u)$. This can be handled exactly as in the $l_2$ set. That is, since *t* never changes, *u* can only be removed from $l_4$ when adding *u* to $Q'$. Thus when adding a vertex *u* to the maximal topological sort, we remove *u* from every $l_4(a, b)$ containing *u*, which takes time $O(n^2)$. Since each *u* gets added at most once in *Q*, maintaining $l_4$ takes $O(n^3)$ times in total.
- (The $l_3$ set). Let $a,b \in X_i$, $i \in \{1,2\}$. Recall that we must have $u \in l_3(a, b)$ if there is $(u, v) \in E(T)$ such that $t(u) = t(v) = \mathfrak {s}$, $\widehat{\mu }_{S'}(u) = x_i$ and $a,b \in \sigma _{T_{\mathcal {\overline{E}}}}(v)$. Note that if $\widehat{\mu }_{S'}(u) = x_i$, then $\widehat{\mu }_{S}(u) = x$, and *x* was the parent of *a* and *b* in *S*. Since *t* does not change after refining, this means that if *u* must be in $l_3(a, b)$ after the binary refinement, then *u* was also in $l_3(a, b)$ before the refinement. It follows that we never add new vertices into $l_3(a, b)$. It therefore suffices to detect when some *u* needs to be removed from $l_3(a, b)$. Recall that when refining *S* into $S'$, $\widehat{\mu }_{S'}(u) \ne \widehat{\mu }_S(u)$ is only possible if $\widehat{\mu }_S(u) =x$ and $\widehat{\mu }_{S'}(u) \in \{x, x_1, x_2\}$. There are thus three cases to consider. If $\widehat{\mu }_{S'}(u) = x$, then we remove *u* from all $l_3(a, b)$ such that $a,b \in X_1$ or $a, b \in X_2$. We note that we will never consider again the $\{a, b, u\}$ triplet because *u* will never be refined again. If $\widehat{\mu }_{S'}(u) = x_1$, then *u* stays present in all $l_3(a, b)$ such that $a,b \in X_1$, but we must remove *u* from every $l_3(a, b)$ such that $a, b \in X_2$. Symmetrically, if $\widehat{\mu }_{S'}(u)$, we remove *u* from every $l_3(a, b)$ such that $a, b \in X_1$. In either case, the triplets $\{a, b, u\}$ evaluated in the last two cases will never be considered again because *u* will never be mapped to an ancestor of *a* and *b* in future refinements of $S'$. Globally, we therefore notice that for any triplet $\{a, b, u\}$ such that $a, b \in L(S)$ and $u \in V(T)$, we only need to consider once the removal of *u* from $l_3(a, b)$, and this removal takes *O*(1) time. We deduce that maintaining $l_3$ requires total time $O(n^3)$.

To summarize, the $l_i$ sets can be kept up-to-date after each split refinement in total time $O(n^3)$. Since the other operations also take time $O(n^3)$, the complete algorithm also takes $O(n^3)$ time.

Finally, among all algorithms that compute $$\mathcal {R}(T; t, \sigma )$$, Lemma 2 implies that the boundary $$O(n^3)$$ is tight, that is the worst-case runtime is $$\Theta (n^3)$$$$\square.$$
